# Supplementary material for: Do hospitalisations for ambulatory care sensitive conditions reflect low access to primary care? An observational cohort study of primary care usage prior to hospitalisation
Source: BMJ Open. 2017 Aug 21;7(8):e015704. doi: 10.1136/bmjopen-2016-015704 (PMC5724125; doi:10.1136/bmjopen-2016-015704)
Supplement: Author's manuscript [file bmjopen-2016-015704.draft_revisions.pdf]

BMJ Open is committed to open peer review. As part of this commitment we make the peer review history of every article we publish publicly available.

When an article is published we post the peer reviewers' comments and the authors' responses online. We also post the versions of the paper that were used during peer review. These are the versions that the peer review comments apply to.

The versions of the paper that follow are the versions that were submitted during the peer review process. They are not the versions of record or the final published versions. They should not be cited or distributed as the published version of this manuscript.

BMJ Open is an open access journal and the full, final, typeset and author-corrected version of record of the manuscript is available on our site with no access controls, subscription charges or pay-per-view fees (http://bmjopen.bmj.com).

If you have any questions on BMJ Open's open peer review process please email editorial.bmjopen@bmj.com

BMJ Open

Do ACSC hospitalisation rates reflect low access to primary care? An observational cohort study of primary care utilisation prior to ACSC hospitalisation

Journal:	*BMJ Open*
Manuscript ID	bmjopen-2016-015704
Article Type:	Research
Date Submitted by the Author:	29-Dec-2016
Complete List of Authors:	Vuik, Sabine; Imperial College London, Institute of Global Health Innovation<br>Fontana, Gianluca; Imperial College London, Institute of Global Health Innovation<br>Mayer, Erik; Imperial College London, Dept. of Surgery & Cancer<br>Darzi, Ara; Imperial College London,
**Primary Subject Heading**:	Health services research
Secondary Subject Heading:	General practice / Family practice
Keywords:	PRIMARY CARE, ACSC, Quality in health care < HEALTH SERVICES ADMINISTRATION & MANAGEMENT, access to care

SCHOLARONE™
Manuscripts

For peer review only

Do ACSC hospitalisation rates reflect low access to primary care? An observational cohort study of primary care utilisation prior to ACSC hospitalisation

Sabine I Vuik, Gianluca Fontana, Erik Mayer, Ara Darzi

Sabine I Vuik, Policy Fellow, Institute of Global Health Innovation
Imperial College, 10th Floor, St Mary's Hospital, Praed Street, London, W1 2NY, UK

Gianluca Fontana, Senior Policy Fellow, Institute of Global Health Innovation
Imperial College, 10th Floor, St Mary's Hospital, Praed Street, London, W1 2NY, UK

Erik Mayer, Clinical Senior Lecturer, Department of Surgery
Imperial College, 10th Floor, St Mary's Hospital, Praed Street, London, W1 2NY, UK

Ara Darzi, Professor in Surgery, Department of Surgery & Institute of Global Health Innovation
Imperial College, 10th Floor, St Mary's Hospital, Praed Street, London, W1 2NY, UK

Correspondence to: Sabine Vuik, s.vuik@imperial.ac.uk
+44(0) 795 714 0479 (no fax available)
Room 1029, 10th Floor, QEQM building, St Mary's Hospital, Praed Street, London, W1 2NY, UK

Keywords: primary care quality, ACSC hospitalisations, integrated care, access to care
Word count (excl. title page, abstract, references, tables and figures): 2,768

For peer review only

ABSTRACT

Objectives To explore whether hospitalisations for Ambulatory Care Sensitive Conditions (ACSCs) are associated with low access to primary care

Design Observational cohort study over 2008 to 2012 using the Clinical Practice Research Datalink (CPRD) and Hospital Episode Statistics (HES) databases

Setting English primary and secondary care

Participants A random sample of 300,000 patients

Main outcome measures Primary care access measured as the number of GP contacts

Results Over the long-term, patients with ACSC hospitalisations had on average 2.33 (2.17 to 2.49) more GP contacts per six months than patients with similar conditions who did not require hospitalisation. When accounting for the number of diagnosed ACSCs, age, gender and GP practice through a nested case-control method, the difference was smaller (0.64 contacts) but still significant ($p<0.001$). People with ACSC hospitalisations also had significantly more outpatient appointments and non-ACSC emergency hospitalisations, but fewer elective day case admissions.

In the six months prior to hospitalisation, patients used more GP services than on average over the five years. Cases had significantly ($p<0.001$) more primary care contacts in the six months before ACSC hospitalisations (7.12, 95% CI: 6.95 to 7.30) than their controls during the same six months (5.57, 95% CI: 5.43 to 5.72). The use of GP services increased closer to the time of hospitalisation, with a peak of 1.79 (1.74 to 1.83) contacts in the last 30 days before hospitalisation.

Conclusions No evidence was found to support the hypothesis that low access to primary care is the main driver of ACSC hospitalisations. Other causes and care settings should be explored to understand how to use ACSC admission rates as quality metrics, and to develop the appropriate interventions.

STRENGTHS AND LIMITATIONS OF THIS STUDY

For peer review only - http://bmjopen.bmj.com/site/about/guidelines.xhtml

- This study uses linked primary and secondary care data to explore whether there exists a patient-level, temporal relation between low utilisation of primary care and ACSC hospitalisations
- It considers both long term (5 years) and short term (6 months prior to hospitalisations) primary care utilisation
- To account for confounders, this study uses a nested case-control design, in addition to looking at average rates in the population
- While primary care utilisation can be considered a proxy of 'realised' access to care, it remains only a proxy and does not consider unmet needs
- This study only looks at access to primary care, and does not explore other quality aspects of primary care that may be related to ACSC hospitalisation, such as timeliness and effectiveness

For peer review only

For peer review only - http://bmjopen.bmj.com/site/about/guidelines.xhtml

BACKGROUND

Ambulatory care sensitive conditions (ACSCs) are conditions like diabetes, COPD and angina that are generally cared for in the community by primary care providers.[1-3] It is thought that high quality primary care, which is timely,[4,5] effective,[4] continuous,[5-7] and accessible,[1,8-14] can prevent emergency hospitalisations for these conditions.[1,15] In addition to capturing a wide range of quality aspects, emergency hospitalisations also reflect an major health system expenditure.[16] For these reasons, the rate of ACSC hospitalisation has been widely used to measure the overall quality of primary care, by national organisations in the UK, the USA, Canada and Australia.[17-20]

Despite the widespread use of these metrics, empirical evidence of their validity is limited.[21] In particular, ACSC hospitalisations are often considered a reflection of access to primary care,[1,8-14,22] but the actual relation between primary care access and hospitalisations for ACSCs remains unclear.[21,23] Various studies have found low access to primary care physicians to be related to higher rates of ACSC hospitalisation.[8-12,14,24] However, these are ecological studies using high-level proxies for primary care access, such as the density of primary care physicians in a certain region.[8-10,24] Other studies have found no relation between primary care access and ACSC hospitalisation rates.[5,25]

Moreover, it is debateable whether the responsibility for preventing ACSC hospitalisations lies solely with primary care. Integrating primary and secondary care, or health and social care, has been shown to reduce emergency hospitalisations.[23,26] Since the care needs for ACSCs may extend beyond primary care, access to other settings can be expected to play a role in the prevention of hospitalisation for these conditions.

Understanding what drives hospitalisations for ACSCs is the first step in developing effective policies to address the issue. This paper aims to identify whether there is a

For peer review only - http://bmjopen.bmj.com/site/about/guidelines.xhtml

relation between low access to primary care and ACSC hospitalisations. Using administrative data linked across primary and secondary care, it explores this relation at a patient-level. It considers both long-term access to primary care, reflecting the long-term management of the condition, and access in the six months prior to a hospitalisation.

METHODS

Study overview

Using a retrospective cohort study, this paper analyses the relation between primary care utilisation and hospitalisations for ACSCs at the patient-level. In addition to exploring population-level rates, matched case-control samples were selected and compared to adjust for a range of confounders. The study looks at both long-term utilisation and utilisation directly prior to ACSC hospitalisation.

To understand the long-term levels of primary care utilisation for patients with ACSC hospitalisations, the average number of GP contacts per six months was calculated from five years of data. To contextualise this utilisation rate and determine whether it is low or high, it was compared to the utilisation rates of two reference populations: patients without any diagnosed ACSCs, and patients with diagnosed ACSCs but no qualifying emergency hospitalisations. *(Note that, for simplicity, the second group is called 'patients with ACSCs but no hospitalisations'. This only refers to qualifying emergency admissions for ACSCs as defined below. Patients in this group may still have had elective hospitalisations, or hospitalisations for non-ACSC causes).* Long-term utilisation rates of other care settings were calculated to understand whether they also relate to hospitalisations for ACSCs.

For peer review only - http://bmjopen.bmj.com/site/about/guidelines.xhtml

To explore whether a temporary lack of primary care contributes to ACSC hospitalisations, primary care utilisation in the six months directly prior to the hospitalisation was also calculated. In addition, utilisation rates for six consecutive 30-day intervals were calculated to study changes in care access leading up to an ACSC hospitalisation.

Data sources and study sample

Using the Clinical Practice Research Datalink (CPRD) and Hospital Episode Statistics (HES), a database containing a random sample of 300,000 English patients and their five-year (2008-2012) primary care and secondary care utilisation was obtained. Patients were eligible for inclusion in the study if they were registered with a CPRD participating GP throughout the entire observation period, and if their primary care data could be linked to their secondary care data in HES. The sample size was set at 300,000, similar to the population of a Clinical Commissioning Group (CCG) in the 75th percentile.[27] This was chosen with the aim of reflecting a typical administrative population, as CCGs in England are the bodies responsible for planning and commissioning care for a local area.

Main outcome measures

The main measures in this study were hospitalisations for ACSCs, and primary care utilisation.

Hospitalisations for ACSC admissions: In the HES data, ACSC hospitalisations were identified following the definitions below. To ensure six months of primary care activity was available preceding each ACSC hospitalisation, only hospitalisations in the final 4.5 years were included.

The definition of ACSCs generally includes both chronic and acute conditions.[28] This study focuses on chronic ACSCs only, as it is interested in long-term disease management in the

For peer review only - http://bmjopen.bmj.com/site/about/guidelines.xhtml

primary care setting. In addition, it allows a comparator group to be created consisting of people with similar chronic conditions but no hospitalisations. A wide variety of ACSC conditions and coding practices exists.[28] This research defines ACSCs according to the ICD-10 codes used in the NHS England Outcomes Framework, as these are specific to English administrative data.[17]

The diagnosis codes for ACSC hospitalisations were also used to identify patients who have been diagnosed with those conditions in the 300,000 patients sample. Patients' morbidity profiles were based on a combination of both the HES and CPRD dataset, with any diagnosis at any point in time being considered as a positive flag for that condition. To be able to find ACSC diagnoses in the CPRD database, the ICD-10 definitions had to be translated to READ codes, which are used in primary care. A mapping created by the NHS Health and Social Care Information Centre was used for this purpose.[29]

Care utilisation: This study aims to measure access to care. While it is difficult to measure 'potential' care access, which includes unmet needs, 'realised' care access can be measured in terms of care utilisation rates.[30] Therefore, utilisation rates are used as a proxy for access. It is important to note that this metric measures the patient's frequency of contact with healthcare providers, but it does not consider other aspects of access such as the length of the contact or instances where a patient is unable to get an appointment.

Utilisation rates were measured for primary care by counting the number of GP contacts, including surgery and clinic attendances, home visits and telephone calls. No distinction was made between different types of practice staff, and care could have been provided by practice nurses and other qualified staff. Therefore, a 'GP contact' should be interpreted as a contact with the GP practice rather than a specific GP.

For peer review only - http://bmjopen.bmj.com/site/about/guidelines.xhtml

For the other care settings, utilisation rates were calculated from HES Outpatient data (the number of outpatient appointments) and HES Inpatient data (the number of elective overnight hospitalisations; the number of elective day case hospitalisations; the number of emergency hospitalisations for non-ACSC causes).

Statistical analysis

Using SPSS Statistics version 23,[31] descriptive statistics, including average utilisation rates, were calculated for the overall population and the three population groups. Independent t-tests were used to compare the utilisation rates in the population with ACSCs hospitalisations to the population with ACSCs but no hospitalisations.

To adjust for timing differences as well as confounders such as age and ACSC counts, a nested case-control method was used. Using the FUZZY extension in SPSS, patients with an ACSC hospitalisation were matched to a control, based on gender, number of diagnosed ACSCs, GP practice (to account for provider-level differences) and ten-year age bands. The long-term primary care utilisation rates, as well as utilisation rates of other care settings, were compared using independent t-tests between the cases and controls. For the utilisation prior to ACSC hospitalisation, the same six-month time period preceding the hospitalisation of the cases was used for the controls, to account for seasonal or other timing differences.

A general linear model for repeated measures was used to compare the different utilisation rates in the six 30-day intervals leading up to an ACSC hospitalisation. These intervals were compared pair-wise as well, adjusting the significance level for multiple tests using a Bonferroni correction.

For peer review only - http://bmjopen.bmj.com/site/about/guidelines.xhtml

RESULTS

In the database, 7,467 hospitalisations for ACSCs were identified for the sample of 300,000 people over 4.5 years. This equates to an admission rate of 553 per 100,000 people per year, which is in line with crude ACSC admission rates for regions in England in 2014/15, which range from 137 to 1,384 per 100,000.[32]

Nearly one-third (65,179) of people in the population had diagnoses for one or more ACSCs (see table 1). Of these people, 8% (5,190) had one or more hospitalisations for their ACSCs, with 4,017 people having one ACSC hospitalisation during the 4.5 years, and 1,173 people more than one.

Table 1: Sample characteristics for the three population groups

	**People with no ACSCs**	**People with ACSCs but no hospitalisations**	**People with hospitalisations for ACSCs**	**Total population**
N	229,631	65,179	5,190	**300,000**
Age (average)	41	58	63	**45**
Gender				
Female (%)	50%	53%	50%	**51%**
Male (%)	50%	47%	50%	**49%**
Number of diagnosed ACSCs (average)	0	1.47	2.60	**0.36**
Number of hospitalisations for ACSCs over 4.5 years (average)	0	0	1.44	**0.02**

For 76% (3,957) of all people with an ACSC hospitalisation an exactly matched control was found (see table 2). This subgroup accounted for 70% of all ACSC hospitalisations in the

For peer review only - http://bmjopen.bmj.com/site/about/guidelines.xhtml

database. While a ten-year age band was used to match people rather than exact age, there was no significant (p=0.62) age difference between the two groups.

Table 2: Sample characteristics of the nested cases and controls

	**Cases**	**Controls**
N	3,957	3,957
Age (average)	62.02	62.27
Gender		
Female (%)	50%	50%
Male (%)	50%	50%
Number of diagnosed ACSCs (average)	2.13	2.13

Long-term primary care utilisation

Over the full study period of five years, patients with ACSC hospitalisations had significantly (p<0.001) more GP contacts per six months (7.48, 95% CI: 7.32 to 7.64) than people with similar conditions who did not require hospitalisation (5.15, 95% CI: 5.12 to 5.18) (see figure 1). The nested case-control method adjusts for the number of diagnosed ACSCs and other confounders. While the difference between these two groups was smaller, people with ACSC hospitalisations still had significantly (p<0.001) more GP contacts than their controls.

In addition to more primary care, the cases also appear to access more outpatient care, and experience more non-ACSC related emergency hospitalisations then their controls (see table 3). However, they had slightly less elective day case care than the controls.

Table 3: Comparison of long-term utilisation of different care services for the matched cases and controls

For peer review only - http://bmjopen.bmj.com/site/about/guidelines.xhtml

	Cases	Controls	Significance of the difference
Number of GP contacts per six months (five year average, 95% CI)	6.79 (6.63 to 6.95)	6.15 (6.01 to 6.30)	p<0.001
Number of outpatient visits per six months (five year average, 95% CI)	2.16 (2.08 to 2.25)	1.79 (1.73 to 1.86)	p<0.001
Number of elective day case admissions per six months (five year average, 95% CI)	0.13 (0.12 to 0.14)	0.15 (0.14 to 0.16)	p=0.013
Number of elective overnight admissions per six months (five year average, 95% CI)	0.05 (0.05 to 0.05)	0.05 (0.05 to 0.06)	p=0.199
Number of non-ACSC emergency hospitalisations per six months (five year average, 95% CI)	0.14 (0.13 to 0.14)	0.10 (0.10 to 0.11)	p<0.001

Primary care utilisation prior to ACSC hospitalisation

Comparing long-term utilisation rates to the utilisation rate immediately prior to an ACSC hospitalisation showed that patients with ACSC hospitalisations saw their GP more often in the six months preceding a hospitalisation (8.19, 95% CI: 8.01 to 8.36) than on average over five years (7.48, 95% CI: 7.32 to 7.64).

For peer review only

As observed for the long-term utilisation, there are factors such as the number of ACSCs that influence primary care utilisation. In addition, since the utilisation directly prior to a hospitalisation is measured over only six months, there may be seasonal differences between this metric and the five-year average rates. Therefore, primary care utilisation prior to hospitalisation in the cases was compared to utilisation in the controls over the same six-month time period. People with ACSC hospitalisations had significantly ($p<0.001$) more primary care contacts in the six months before hospitalisations (7.12, 95% CI: 6.95 to 7.30) than their controls during the same six months (5.57, 95% CI: 5.43 to 5.72).

To explore changes in primary care use leading up to an ACSC hospitalisation, the utilisation rates of six consecutive 30-day intervals were calculated (see figure 2). GP contacts were skewed towards the months closer to the hospitalisation date. A repeated measures general linear model showed that the mean number of GP contacts is significant different ($p<0.001$) across the six 30-day intervals. Pairwise comparisons show that the peak in GP contacts in the final 30 days prior to hospitalisation is significantly different from each of the five preceding months. *(Note: the sum of these intervals (8.11) is slightly less than the six-month utilisation (8.19), as the latter is calculated over 182 days rather than 180 to reflect 6 calendar months, allowing comparison to the 5-year utilisation rates).*

DISCUSSION

The primary objective of this study was to explore whether low access to primary care is related to hospitalisations for ACSCs. No evidence was found of low primary care utilisation either over the long term, which could affect the on-going management of ACSCs, or in the short term, prior to an ACSC hospitalisation. The results show that, over five years, patients with ACSC hospitalisations in fact use more GP services compared to other patients with the same number of diagnosed ACSCs, age, gender and GP practice.

For peer review only - http://bmjopen.bmj.com/site/about/guidelines.xhtml

Neither does there appear to be a temporary drop in utilisation. In the six months immediately prior to an ACSC hospitalisation, utilisation is higher than the five-year average. People with ACSC hospitalisations use more primary care than matched controls over the same six-month period. In addition, the average utilisation rate increased every month nearer to the hospitalisation, with a significant peak in the last 30 days prior to hospitalisation. Overall, this research finds no evidence that low access to GP services is a key driver of ACSC hospitalisation.

It is debateable whether the responsibility for preventing ACSC hospitalisations lies solely with primary care. The results show that, in addition to primary care, utilisation of elective inpatient and outpatient care is also significantly different for people with ACSC hospitalisations. This should be taken into account when developing interventions for this population, though further research is needed to understand the interplay between different care settings. Population segmentation could help to identify different care utilisation patterns.

This study found a statistically significant relation between primary care utilisation and ACSC hospitalisations, where the utilisation rate increases prior to hospitalisation. Future research should explore whether this correlation could be used to improve risk models that focus on prediction emergency admissions.

An important strength of this study is the use of linked, administrative data at the patient level. Rather than using population-level proxies for access, as has been done in previous studies on this topic,[8-10,24] this study linked primary care utilisation and ACSC hospitalisations at the patient level. In addition, it considered both long-term care use and care utilisation immediately preceding an ACSC hospitalisation. Using this approach, the

For peer review only - http://bmjopen.bmj.com/site/about/guidelines.xhtml

For peer review only

direct, temporal relation between a patient's access to primary care and ACSC hospitalisation could be explored.

The research has a number of limitations, in addition to the well-documented general limitations associated with using administrative healthcare data.[33] Firstly, while utilisation approximates access to care at the patient level, it is still only a proxy. No differentiation can be made between low utilisation due to limited access to services, and other causes such as patients not seeking care. In addition, this proxy does not take into account any unmet needs. Utilisation rates for patients with ACSC hospitalisations were in line with those of people with similar disease profiles, but their actual need for care may have been even higher - as suggested in their eventual hospitalisation. The fact that ACSC hospitalisations are not associated with lower access to primary care does not preclude that more primary care could prevent them.

Secondly, this paper only explores the relation between low access to primary care and ACSC hospitalisations. ACSC hospitalisation rates are used as a high-level measure of primary care, and can been linked to many other aspects of care quality, such as timeliness,[4,5] effectiveness,[4] continuity,[5-7] safety and appropriateness.[34] Further research is needed to understand the specific and relative impact of these factors on ACSC hospitalisations.

Thirdly, there remains the possibility of confounding. While a nested case-control method was used to account for diagnosed ACSCs, age, gender and provider differences, other factors such as ethnicity[22] and socio-economic status[5,35] could be influencing the results. The cases for which a matched control was found had slightly less primary care activity and ACSC hospitalisations than the overall population with ACSC hospitalisations. This

For peer review only - http://bmjopen.bmj.com/site/about/guidelines.xhtml

could be due to a lack of matched controls for extreme cases with a very high age or ACSCs count, who could also be expected to use more care.

The results presented here have important implications for clinicians and policymakers. This study suggests that, contrary to common belief, low access to primary care is not a leading cause of ACSC hospitalisations. This would mean that ACSC hospitalisation rates, while informative, should not be used specifically as indicators of access to primary care.

Moreover, policymakers and clinicians trying to reduce emergency hospitalisation should consider interventions that focus on other aspects of primary care, such as effectiveness and continuity of care. In addition, they should take into account the impact that other care settings may have on ACSC hospitalisations, and focus on integrating care across the continuum rather than on primary care alone.

CONCLUSION

This research found no patient-level, temporal relation between low access to primary care services and hospitalisations for ACSCs. While there may be other aspects of primary care quality that impact ACSC hospitalisation rates, low access does not appear to be a key driver. More research is needed into the actual causes of ACCS hospitalisations, across care settings, to allow accurate interpretation of ACSC hospitalisation rates and to develop effective interventions.

FIGURE LEGENDS

For peer review only - http://bmjopen.bmj.com/site/about/guidelines.xhtml

Figure 1: Average number of GP contacts per six months

Figure 2: Average number of GP contacts prior to an ACSC hospitalisation

For peer review only

For peer review only - http://bmjopen.bmj.com/site/about/guidelines.xhtml

STATEMENTS

Database: This study is based on data from the Clinical Practice Research Datalink obtained under license from the UK Medicines and Healthcare Products Regulatory Agency. However, the interpretation and conclusions contained in the study are those of the authors alone. The study was approved under reference number 14_211RA.

Data sharing: The SPSS syntax documents detailing the steps taken to create the analysis databases from raw CPRD and HES data are available upon request from the corresponding author.

Funding: The research was funded by the National Institute for Health Research (NIHR) Biomedical Research Centre based at Imperial College Healthcare NHS Trust and Imperial College London. The views expressed are those of the authors and not necessarily those of the NHS, the NIHR or the Department of Health.

This project was also supported by the Peter Sowerby Foundation.

Competing interests: The authors have no competing interests

Ethics approval: No ethics approval was required

Patient involvement: No patients were involved in this study

Transparency declaration: The authors affirm that the manuscript is an honest, accurate, and transparent account of the study being reported; that no important aspects of the study have been omitted; and that any discrepancies from the study as planned have been explained.

For peer review only

Contributors: SV designed the study, created the database, analysed the data, and drafted and revised the paper. She is guarantor. GF and EM contributed to the design of the study, analysed the results and revised the draft paper. AD contributed to the design of the study and revised the draft paper. All have approved the final version for publication.

Exclusive license: The Corresponding Author has the right to grant on behalf of all authors and does grant on behalf of all authors, a worldwide licence to the Publishers and its licensees in perpetuity, in all forms, formats and media (whether known now or created in the future), to i) publish, reproduce, distribute, display and store the Contribution, ii) translate the Contribution into other languages, create adaptations, reprints, include within collections and create summaries, extracts and/or, abstracts of the Contribution and convert or allow conversion into any format including without limitation audio, iii) create any other derivative work(s) based in whole or part on the on the Contribution, iv) to exploit all subsidiary rights to exploit all subsidiary rights that currently exist or as may exist in the future in the Contribution, v) the inclusion of electronic links from the Contribution to third party material where-ever it may be located; and, vi) licence any third party to do any or all of the above.

A **RECORD checklist** has been completed for this study. "REporting of studies Conducted using Observational Routinely-collected Data" (RECORD) extends the STROBE guidelines to studies using administrative data.

For peer review only

REFERENCES

1. Billings J, Anderson GM, Newman LS. Recent findings on preventable hospitalizations. Health Aff (Millwood) 1996;**15**(3):239-49.

2. Bindman AB, Grumbach K, Osmond D, et al. Preventable hospitalizations and access to health care. JAMA 1995;**274**(4):305-11.

3. Billings J, Zeitel L, Lukomnik J, et al. Impact of socioeconomic status on hospital use in New York City. Health Aff (Millwood) 1993;**12**(1):162-73.

4. Caminal J, Starfield B, Sánchez E, et al. The role of primary care in preventing ambulatory care sensitive conditions. The European Journal of Public Health 2004;**14**(3):246-51.

5. Magán P, Alberquilla Á, Otero Á, et al. Hospitalizations for Ambulatory Care Sensitive Conditions and Quality of Primary Care: Their Relation With Socioeconomic and Health Care Variables in the Madrid Regional Health Service (Spain). Med Care 2011;**49**(1):17-23.

6. Menec VH, Sirski M, Attawar D, et al. Does continuity of care with a family physician reduce hospitalizations among older adults? J Health Serv Res Policy 2006;**11**(4):196-201.

7. Lambrew J, Defriese G, Carey T, et al. The Effects of Having a Regular Doctor on Access to Primary Care. Med Care 1996;**34**(2):138-51.

8. Laditka JN, Laditka SB, Probst JC. More May Be Better: Evidence of a Negative Relationship between Physician Supply and Hospitalization for Ambulatory Care Sensitive Conditions. Health Serv Res 2005;**40**(4):1148-66.

9. Ansari Z, Laditka JN, Laditka SB. Access to Health Care and Hospitalization for Ambulatory Care Sensitive Conditions. Med Care Res Rev 2006;**63**(6):719-41.

10. Basu J, Friedman B, Burstin H. Primary Care, HMO Enrollment, and Hospitalization for Ambulatory Care Sensitive Conditions: A New Approach. Med Care 2002;**40**(12):1260-69.

11. Macinko J, de Oliveira VB, Turci MA, et al. The Influence of Primary Care and Hospital Supply on Ambulatory Care–Sensitive Hospitalizations Among Adults in Brazil, 1999–2007. Am J Public Health 2011;**101**(10):1963-70.

12. Rizza P, Bianco A, Pavia M, et al. Preventable hospitalization and access to primary health care in an area of Southern Italy. BMC Health Serv Res 2007;**7**(1):1-8.

13. Freund T, Campbell SM, Geissler S, et al. Strategies for Reducing Potentially Avoidable Hospitalizations for Ambulatory Care–Sensitive Conditions. The Annals of Family Medicine 2013;**11**(4):363-70.

14. Probst JC, Laditka JN, Laditka SB. Association between community health center and rural health clinic presence and county-level hospitalization rates for ambulatory care sensitive conditions: an analysis across eight US states. BMC Health Serv Res 2009;**9**(1):1-11.

For peer review only

For peer review only - http://bmjopen.bmj.com/site/about/guidelines.xhtml

15. Blunt I. Focus on preventable admissions - Trends in emergency admissions for ambulatory care sensitive conditions, 2001 to 2013. Quality Watch. London: The Health Foundation & Nuffield Trust, 2013.

16. Morse A. Emergency admissions to hospital: managing the demand. London: National Audit Office, 2013.

17. NHS Information Centre for Health and Social Care. NHS Outcomes Framework 2014/15: Domain 2 Enhancing quality of life for people with long-term conditions - Indicator specifications. Leeds: NHS Information Centre for Health and Social Care, 2014.

18. Agency for Healthcare Research and Quality. Prevention Quality Indicators Overview. Secondary Prevention Quality Indicators Overview. http://www.qualityindicators.ahrq.gov/modules/pqi_overview.aspx.

19. Canadian Institute for Health Information. Technical Note: Ambulatory Care Sensitive Conditions (ACSCs) Hospitalization Rate. Secondary Technical Note: Ambulatory Care Sensitive Conditions (ACSCs) Hospitalization Rate. https://www.cihi.ca/en/health-system-performance/performance-reporting/indicators/technical-note-ambulatory-care-sensitiv-0.

20. Glover J, Page A, Ambrose S, et al. Atlas of Avoidable Hospitalisations in Australia: ambulatory care-sensitive conditions. Canberra: Australian Institute of Health and Welfare, 2007.

21. Solberg LI. Preventable hospital admissions: are they? Family Practice 2015;**32**(3):245-46.

22. Laditka JN, Laditka SB, Mastanduno MP. Hospital utilization for ambulatory care sensitive conditions: health outcome disparities associated with race and ethnicity. Social science & medicine 2003;**57**(8):1429-41.

23. Purdy S. Avoiding hospital admissions - What does the research evidence say? London: The Kings Fund, 2010.

24. Gibson OR, Segal L, McDermott RA. A systematic review of evidence on the association between hospitalisation for chronic disease related ambulatory care sensitive conditions and primary health care resourcing. BMC Health Serv Res 2013;**13**(1):1-13.

25. Ricketts TC, Randolph R, Howard HA, et al. Hospitalization rates as indicators of access to primary care. Health & Place 2001;**7**(1):27-38.

26. Torjesen I. Emergency admissions for diabetes fall by almost 7% in integrated care pilot scheme. Bmj 2012;**344**.

27. NHS Health and Social Care Information Centre. Numbers of Patients Registered at a GP Practice - July 2015. Leeds: NHS Health and Social Care Information Centre, 2015.

For peer review only

28. Purdy S, Griffin T, Salisbury C, et al. Ambulatory care sensitive conditions: terminology and disease coding need to be more specific to aid policy makers and clinicians. Public Health 2009;**123**(2):169-73.

29. Technology Reference Data Update Distribution Service. NHS UK Read Codes Clinical Terms Version 3. 18.0.2_20141001000001 ed. Leeds: NHS Information Centre for Health and Social Care, 2014.

30. Goddard M, Smith P. Equity of access to health care services: Theory and evidence from the UK. Social science & medicine 2001;**53**(9):1149-62.

31. IBM SPSS Statistics for Macintosh, Version 23.0 [program]. Armonk, NY: IBM Corp, 2015.

32. NHS Health and Social Care Information Centre. CCG OIS 2.6 Unplanned hospitalisation for chronic ambulatory care sensitive conditions. December 2015 ed, 2015.

33. Virnig BA, McBean M. Administrative Data for Public Health Surveillance and Planning. Annual Review of Public Health 2001;**22**(1):213-30.

34. Arah OA, Westert GP, Hurst J, et al. A conceptual framework for the OECD Health Care Quality Indicators Project. Int J Qual Health Care 2006;**18**(suppl 1):5-13.

35. Roos LL, Walld R, Uhanova J, et al. Physician Visits, Hospitalizations, and Socioeconomic Status: Ambulatory Care Sensitive Conditions in a Canadian Setting. Health Serv Res 2005;**40**(4):1167-85.

For peer review only

For peer review only - http://bmjopen.bmj.com/site/about/guidelines.xhtml

Average number of GP contacts per six months

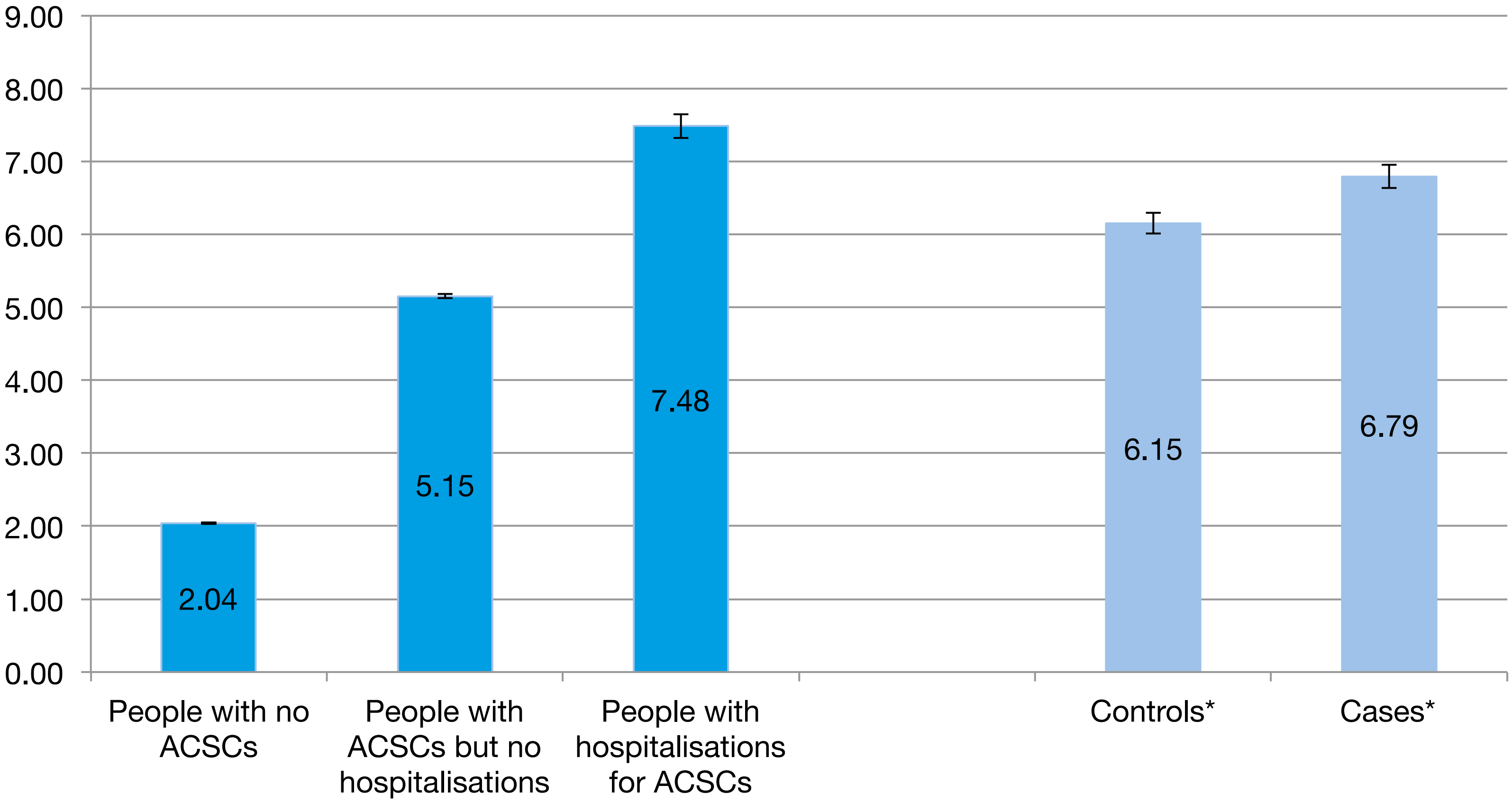

** Samples matched on ten-year age band, number of diagnosed ACSCs, GP practice and gender*

95% confidence interval

Average number of GP contacts prior to an ACSC hospitalisation

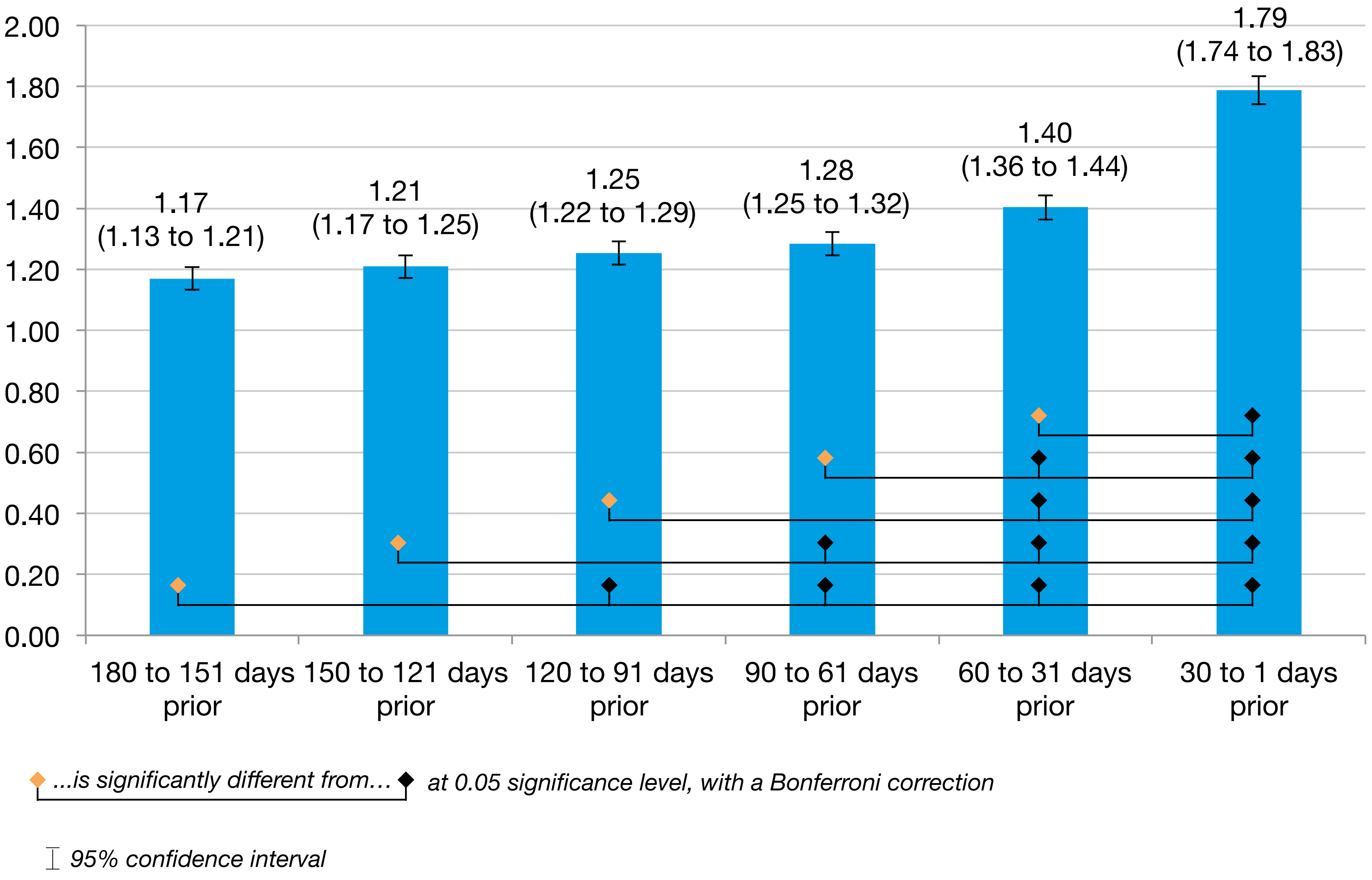

BMJ Open

Do hospitalisations for Ambulatory Care Sensitive Conditions reflect low access to primary care? An observational cohort study of primary care utilisation prior to hospitalisation

Journal:	*BMJ Open*
Manuscript ID	bmjopen-2016-015704.R1
Article Type:	Research
Date Submitted by the Author:	12-May-2017
Complete List of Authors:	Vuik, Sabine; Imperial College London, Institute of Global Health Innovation<br>Fontana, Gianluca; Imperial College London, Institute of Global Health Innovation<br>Mayer, Erik; Imperial College London, Dept. of Surgery & Cancer<br>Darzi, Ara; Imperial College London,
**Primary Subject Heading**:	Health services research
Secondary Subject Heading:	General practice / Family practice
Keywords:	PRIMARY CARE, ACSC, Quality in health care < HEALTH SERVICES ADMINISTRATION & MANAGEMENT, access to care

SCHOLARONE™
Manuscripts

For peer review only

Do hospitalisations for Ambulatory Care Sensitive Conditions reflect low access to primary care? An observational cohort study of primary care utilisation prior to hospitalisation

Sabine I Vuik, Gianluca Fontana, Erik Mayer, Ara Darzi

Sabine I Vuik, Policy Fellow, Institute of Global Health Innovation

Imperial College, 10th Floor, St Mary's Hospital, Praed Street, London, W1 2NY, UK

Gianluca Fontana, Senior Policy Fellow, Institute of Global Health Innovation

Imperial College, 10th Floor, St Mary's Hospital, Praed Street, London, W1 2NY, UK

Erik Mayer, Clinical Senior Lecturer, Department of Surgery

Imperial College, 10th Floor, St Mary's Hospital, Praed Street, London, W1 2NY, UK

Ara Darzi, Professor in Surgery, Department of Surgery & Institute of Global Health Innovation

Imperial College, 10th Floor, St Mary's Hospital, Praed Street, London, W1 2NY, UK

For peer review only

For peer review only - http://bmjopen.bmj.com/site/about/guidelines.xhtml

Correspondence to: Sabine Vuik, s.vuik@imperial.ac.uk

+44(0) 795 714 0479 (no fax available)

Room 1029, 10th Floor, QEQM building, St Mary's Hospital, Praed Street, London, W1 2NY, UK

For peer review only

Keywords: primary care quality, ACSC hospitalisations, integrated care, access to care

Word count (excl. title page, abstract, references, tables and figures): 2,768

ABSTRACT

Objectives To explore whether hospitalisations for Ambulatory Care Sensitive Conditions (ACSCs) are associated with low access to primary care

Design Observational cohort study over 2008 to 2012 using the Clinical Practice Research Datalink (CPRD) and Hospital Episode Statistics (HES) databases

Setting English primary and secondary care

Participants A random sample of 300,000 patients

Main outcome measures Emergency hospitalisation for an ACSC

Results Over the long-term, patients with ACSC hospitalisations had on average 2.33 (2.17 to 2.49) more GP contacts per six months than patients with similar conditions who did not require hospitalisation. When accounting for the number of diagnosed ACSCs, age, gender and GP practice through a nested case-control method, the difference was smaller (0.64 contacts) but still significant ($p<0.001$). People with ACSC hospitalisations also had significantly more outpatient appointments and non-ACSC emergency hospitalisations, but fewer elective day case admissions.

In the short-term, measured over the six months prior to hospitalisation, patients used more GP services than on average over the five years. Cases had significantly ($p<0.001$) more primary care contacts in the six months before ACSC hospitalisations (7.12, 95% CI: 6.95 to 7.30) than their controls during the same six months (5.57, 95% CI: 5.43 to 5.72). The use of GP services increased closer to the time of hospitalisation, with a peak of 1.79 (1.74 to 1.83) contacts in the last 30 days before hospitalisation.

Conclusions This study found no evidence to support the hypothesis that low access to primary care is the main driver of ACSC hospitalisations. Other causes should also be explored to understand how to use ACSC admission rates as quality metrics, and to develop the appropriate interventions.

STRENGTHS AND LIMITATIONS OF THIS STUDY

For peer review only - http://bmjopen.bmj.com/site/about/guidelines.xhtml

- This study uses linked primary and secondary care data to explore whether there exists a patient-level, temporal relation between low utilisation of primary care and ACSC hospitalisations
- It considers both long term (5 years) and short term (6 months prior to hospitalisations) primary care utilisation
- To account for confounders, this study uses a nested case-control design, in addition to looking at average rates in the population
- While primary care utilisation can be considered a proxy of 'realised' access to care, it remains only a proxy and does not consider unmet needs
- This study only looks at access to primary care, and does not explore other quality aspects of primary care that may be related to ACSC hospitalisation, such as timeliness and effectiveness

For peer review only

BACKGROUND

Chronic ambulatory care sensitive conditions (ACSCs) are conditions like diabetes, COPD and angina that are cared for in the community by primary care providers.[1-3] With high quality primary care - which is timely,[4,5] effective,[4] continuous,[5-8] and accessible[1,9-15] - these conditions generally should not require hospital care. Therefore, emergency hospitalisations for chronic ACSCs can be considered a failing of primary care.[1,16,17]

In addition to providing a measure of low care quality, emergency hospitalisations for ACSC also reflect a major health system expenditure,[18] and a negative patient experience - covering all elements of the Triple Aim.[19] As a result, the rate of ACSC hospitalisation has been widely used to measure the overall quality of primary care, by national organisations in the UK, the USA, Canada and Australia.[20-23]

Despite the widespread use of these metrics, empirical evidence of their validity is limited.[24] In particular, ACSC hospitalisations are often considered a reflection of access to primary care,[1,9-15,25] but the actual relation between primary care access and hospitalisations for ACSCs remains unclear.[24,26] Various studies have found low access to primary care physicians to be related to higher rates of ACSC hospitalisation.[9-13,15,27] However, these are ecological studies using high-level proxies for primary care access, such as the density of primary care physicians in a certain region.[9-11,27] Other studies have found no relation between primary care access and ACSC hospitalisation rates.[5,28,29]

Understanding what drives hospitalisations for ACSCs is the first step in developing effective policies to address the issue. This paper aims to identify whether there is a relation between low access to primary care and ACSC hospitalisations. Using administrative data linked across primary and secondary care, it explores this relation at a

For peer review only

patient-level. It considers both long-term access to primary care, reflecting the long-term management of the condition, and access in the six months prior to a hospitalisation.

METHODS

Study overview

Using a retrospective cohort study, this paper analyses the relation between primary care utilisation and hospitalisations for ACSCs at the patient-level. In addition to exploring population-level rates, matched case-control samples were selected and compared to adjust for a range of confounders. The study looks at both long-term utilisation, as measured over five years, and utilisation directly prior to ACSC hospitalisation, as measured over the six months preceding hospitalisation.

To understand the long-term levels of primary care utilisation for patients with ACSC hospitalisations, the average number of GP contacts per six months was calculated from five years of data. To contextualise this utilisation rate and determine whether it is low or high, it was compared to the utilisation rates of two reference populations: patients without any diagnosed ACSCs, and patients with diagnosed ACSCs but no qualifying emergency hospitalisations. *(Note that, for simplicity, the second group is called 'patients with ACSCs but no hospitalisations'. This only refers to qualifying emergency admissions for ACSCs as defined below. Patients in this group may still have had elective hospitalisations, or hospitalisations for non-ACSC causes).*

To explore whether a temporary lack of primary care contributes to ACSC hospitalisations, primary care utilisation in the six months directly prior to the hospitalisation was also calculated. In addition, utilisation rates for six consecutive 30-day intervals were calculated to study changes in care access leading up to an ACSC hospitalisation. These retrospective

For peer review only - http://bmjopen.bmj.com/site/about/guidelines.xhtml

analyses were done at the ACSC hospitalisation level rather than the patient level by identifying the six months of GP utilisation prior to each individual ACSC hospitalisation. All hospitalisations were included, even if they occurred in the same person, or if their six months of prior activity overlapped with another ACSC hospitalisation for that patient.

Data sources and study sample

Using the Clinical Practice Research Datalink (CPRD) and Hospital Episode Statistics (HES), a database containing a random sample of 300,000 English patients and their five-year (2008-2012) primary care and secondary care utilisation was created. Patients were eligible for inclusion in the study if they were registered with a CPRD participating GP throughout the entire observation period, and if their primary care data could be linked to their secondary care data in HES. Linkage of the data was done by CPRD, and required consent and the availability of matching NHS number and other personal details (note: this information was not available to the authors, who only had anonymous data). The sample size was set at 300,000, which is the maximum sample size CPRD ordinarily allows.

Main outcome measures

The main measures in this study were hospitalisations for ACSCs, and primary care utilisation.

Hospitalisations for ACSC admissions: In the HES data, ACSC hospitalisations were identified following the definitions below. To ensure six months of primary care activity was available preceding each ACSC hospitalisation, only hospitalisations in the final 4.5 years were included.

The definition of ACSCs generally includes both chronic and acute conditions.[30] This study focuses on chronic ACSCs only, as it is interested in long-term disease management in the

For peer review only - http://bmjopen.bmj.com/site/about/guidelines.xhtml

primary care setting. In addition, it allows a comparator group to be created consisting of people with similar chronic conditions but no hospitalisations. A wide variety of ACSC conditions and coding practices exists.[30] This research defines ACSCs according to the ICD-10 codes used in the NHS England Outcomes Framework, as these are specific to English administrative data.[20]

The diagnosis codes for ACSC hospitalisations were also used to identify patients who have been diagnosed with those conditions in the 300,000 patients sample. Patients' morbidity profiles were based on a combination of both the HES and CPRD dataset. Without data outside the five-year study period, we were unable to identify whether a diagnosis code at a certain time point is a newly onset disease, or reflects treatment for an existing condition. Therefore, no temporal analysis was possible, and a diagnosis at any time point was treated as a positive flag for that condition. To be able to find ACSC diagnoses in the CPRD database, the ICD-10 definitions had to be translated to READ codes, which are used in primary care. A mapping created by the NHS Health and Social Care Information Centre was used for this purpose.[31]

Care utilisation: This study aims to measure access to care. While it is difficult to measure 'potential' care access, which includes unmet needs, 'realised' care access can be measured in terms of care utilisation rates.[32] Therefore, utilisation rates are used as a proxy for access. It is important to note that this metric measures the patient's frequency of contact with healthcare providers, but it does not consider other aspects of access such as the length of the contact or instances where a patient is unable to get an appointment.

Utilisation rates were measured for primary care by counting the number of GP contacts, including surgery and clinic attendances, home visits and telephone calls. No distinction was made between different types of practice staff, and care could have been provided by

For peer review only - http://bmjopen.bmj.com/site/about/guidelines.xhtml

practice nurses and other qualified staff. Therefore, a 'GP contact' should be interpreted as a contact with the GP practice rather than a specific GP.

Statistical analysis

Using SPSS Statistics version 23,[33] descriptive statistics, including average utilisation rates, were calculated for the overall population and the three population groups. Independent t-tests were used to compare the utilisation rates in the population with ACSCs hospitalisations to the population with ACSCs but no hospitalisations.

To adjust for timing differences as well as confounders such as age and ACSC counts, a nested case-control method was used. Using the FUZZY extension in SPSS, patients with an ACSC hospitalisation were matched to a control, based on gender, number of diagnosed ACSCs, GP practice (to account for provider-level differences) and ten-year age bands. The long-term primary care utilisation rates, as well as utilisation rates of other care settings, were compared using independent t-tests between the cases and controls. For the utilisation prior to ACSC hospitalisation, the same six-month time period preceding the hospitalisation of the cases was used for the controls, to account for seasonal or other timing differences.

To analyse the change in utilisation rate leading up to an ACSC hospitalisation, a general linear model for repeated measures was used with the utilisation rates for the six 30-day intervals as the response variable. These intervals were compared pair-wise as well, adjusting the significance level for multiple tests using a Bonferroni correction.

RESULTS

For peer review only - http://bmjopen.bmj.com/site/about/guidelines.xhtml

In the database, 7,467 hospitalisations for ACSCs were identified for the sample of 300,000 people over 4.5 years. This equates to an admission rate of 553 per 100,000 people per year, which is in line with crude ACSC admission rates for regions in England in 2014/15, which range from 137 to 1,384 per 100,000.[34]

Nearly one-third (65,179) of people in the population had diagnoses for one or more ACSCs (see table 1). Of these people, 8% (5,190) had one or more hospitalisations for their ACSCs, with 4,017 people having one ACSC hospitalisation during the 4.5 years, and 1,173 people more than one.

Table 1: Sample characteristics for the three population groups

	**People with no ACSCs**	**People with ACSCs but no hospitalisations**	**People with hospitalisations for ACSCs**	**Total population**
N	229,631	65,179	5,190	**300,000**
Age (average)	41	58	63	**45**
Gender				
Female (%)	50%	53%	50%	**51%**
Male (%)	50%	47%	50%	**49%**
Number of diagnosed ACSCs (average)	0	1.47	2.60	**0.36**
Number of hospitalisations for ACSCs over 4.5 years (average)	0	0	1.44	**0.02**

For 76% (3,957) of all people with an ACSC hospitalisation an exactly matched control was found (see table 2). This subgroup accounted for 70% of all ACSC hospitalisations in the

For peer review only - http://bmjopen.bmj.com/site/about/guidelines.xhtml

database. While a ten-year age band was used to match people rather than exact age, there was no significant (p=0.62) age difference between the two groups.

Table 2: Sample characteristics of the nested cases and controls

	**Cases**	**Controls**
N	3,957	3,957
Age (average)	62.02	62.27
Gender		
Female (%)	50%	50%
Male (%)	50%	50%
Number of diagnosed ACSCs (average)	2.13	2.13

Over the full study period of five years, patients with ACSC hospitalisations had significantly more (2.33, p<0.001) GP contacts per six months (7.48, 95% CI: 7.32 to 7.64) than people with similar conditions who did not require hospitalisation (5.15, 95% CI: 5.12 to 5.18) (see figure 1). The nested case-control method adjusts for the number of diagnosed ACSCs and other confounders. While the difference between these two groups was smaller, people with ACSC hospitalisations still had significantly more (0.64, p<0.001) GP contacts than their controls.

Since the utilisation directly prior to a hospitalisation is measured over only six months, there may be seasonal differences for this metric. Therefore, primary care utilisation prior to hospitalisation in the cases was compared to utilisation in the controls over the same six-month time period. People with ACSC hospitalisations had significantly more (1.55, p<0.001) primary care contacts in the six months before hospitalisations (7.12, 95% CI: 6.95 to 7.30) than their controls during the same six months (5.57, 95% CI: 5.43 to 5.72).

For peer review only - http://bmjopen.bmj.com/site/about/guidelines.xhtml

To explore differences in primary care use leading up to ACSC hospitalisations, the utilisation rates of six consecutive 30-day intervals were calculated (see figure 2). GP contacts were skewed towards the months closer to the hospitalisation date. A repeated measures general linear model showed that the mean number of GP contacts is significant different ($p<0.001$) across the six 30-day intervals. Pairwise comparisons show that the peak in GP contacts in the final 30 days prior to hospitalisation is significantly different from each of the five preceding months. *(Note: the sum of these intervals (8.11) is slightly less than the six-month utilisation (8.19), as the latter is calculated over 182 days rather than 180 to reflect 6 calendar months, allowing comparison to the 5-year utilisation rates).*

DISCUSSION

The primary objective of this study was to explore whether low access to primary care is related to hospitalisations for ACSCs. No evidence was found of low primary care utilisation either over the long term, which could affect the on-going management of ACSCs, or in the short term, prior to an ACSC hospitalisation. The results show that, over five years, patients with ACSC hospitalisations in fact use more GP services compared to other patients with the same number of diagnosed ACSCs, age, gender and GP practice.

Neither does there appear to be a temporary drop in utilisation. In the six months immediately prior to an ACSC hospitalisation, utilisation is higher than the five-year average. People with ACSC hospitalisations use more primary care than matched controls over the same six-month period. In addition, the average utilisation rate increased every month nearer to the hospitalisation, with a significant peak in the last 30 days prior to hospitalisation. Overall, this research finds no evidence that low access to GP services is a key driver of ACSC hospitalisation.

For peer review only - http://bmjopen.bmj.com/site/about/guidelines.xhtml

This study found a statistically significant relation between primary care utilisation and ACSC hospitalisations, where the utilisation rate increases prior to hospitalisation. Future research should explore whether this correlation can be used to improve risk models. Predictive models could be developed that predict ACSC hospitalisations based on a change in GP utilisation, allowing preventive interventions to be delivered.

An important strength of this study is the use of linked, administrative data at the patient level. Rather than using population-level proxies for access, as has been done in previous studies on this topic,[9-11,27] this study linked primary care utilisation and ACSC hospitalisations at the patient level. In addition, it considered both long-term care use and care utilisation immediately preceding an ACSC hospitalisation. Using this approach, the direct, temporal relation between a patient's access to primary care and ACSC hospitalisation could be explored.

The research has a number of limitations, in addition to the well-documented general limitations associated with using administrative healthcare data.[35] Firstly, while utilisation approximates access to care at the patient level, it is still only a proxy. No differentiation can be made between low utilisation due to limited access to services, and other causes such as patients not seeking care. In addition, this proxy does not take into account any unmet needs. Utilisation rates for patients with ACSC hospitalisations were in line with those of people with similar disease profiles, but their actual need for care may have been even higher - as suggested in their eventual hospitalisation. The fact that ACSC hospitalisations are not associated with lower access to primary care does not preclude that more primary care could prevent them.

This is a crucial limitation of this research, and of any similar analyses based on administrative data. To fully understand what drives ACSC hospitalisations at the individual

For peer review only - http://bmjopen.bmj.com/site/about/guidelines.xhtml

patient-level, case note reviews or interviews are needed. Administrative data only covers actualised care, while patients are able to provide information on unmet needs, the reasons behind this, and the impact it has had.

Secondly, this paper only explores the relation between low access to primary care and ACSC hospitalisations. ACSC hospitalisation rates are used as a high-level measure of primary care, and can been linked to many other aspects of care quality, such as timeliness,[4,5] effectiveness,[4] continuity,[5-7] safety and appropriateness.[36] Further research is needed to understand the specific and relative impact of these factors on ACSC hospitalisations.

Thirdly, there remains the possibility of confounding. While a nested case-control method was used to account for diagnosed ACSCs, age, gender and provider differences, other factors such as urban/rural environments, ethnicity[25] and socio-economic status[5,37] could be influencing the results. While matching on GP practice partially mitigates these issues due to patients living in the same geographic area, there remains room for confounding.

In addition, cases were matched based on the number of ACSCs rather than specific conditions - limiting the comparability of the cases and controls. This approach was chosen to ensure more matches could be found, and to create a quantitative measure of health status. Nevertheless, health status remains an important potential confounder, particularly due to its direct impact on care utilisation. This is another area where case reviews would provide a level of detail that is not available in administrative data.

The cases for which a matched control was found had slightly less primary care activity and ACSC hospitalisations than the overall population with ACSC hospitalisations. This

For peer review only - http://bmjopen.bmj.com/site/about/guidelines.xhtml

could be due to a lack of matched controls for extreme cases with a very high age or ACSCs count, who could also be expected to use more care.

The results presented here have important implications for clinicians and policymakers. This study provides no evidence that, as is commonly believed, low access to primary care is a leading cause of ACSC hospitalisations. This would mean that ACSC hospitalisation rates, while informative, should not be used specifically as indicators of access to primary care.

More research is needed into new models of care to meet the needs of patients with ACSC hospitalisations. Policymakers and clinicians trying to reduce emergency hospitalisation should consider policies that focus on a variety of aspects of primary care, including effectiveness and continuity of care, rather than focusing on access alone. Tailored interventions aimed at high-risk patients should also be explored, including remote support and observational beds.

CONCLUSION

This research found no patient-level, temporal relation between low utilisation of primary care services and hospitalisations for ACSCs. While there may be other aspects of primary care quality that impact ACSC hospitalisation rates, there is no evidence that low access to care is a key driver. More research is needed into the actual causes of ACCS hospitalisations, across care settings, to allow accurate interpretation of ACSC hospitalisation rates and to develop effective interventions.

For peer review only - http://bmjopen.bmj.com/site/about/guidelines.xhtml
For peer review only

FIGURE LEGENDS

Figure 1: Average number of GP contacts per six months

Figure 2: Average number of GP contacts prior to an ACSC hospitalisation

For peer review only

For peer review only - http://bmjopen.bmj.com/site/about/guidelines.xhtml

STATEMENTS

Database: This study is based on data from the Clinical Practice Research Datalink obtained under license from the UK Medicines and Healthcare Products Regulatory Agency. However, the interpretation and conclusions contained in the study are those of the authors alone. The study was approved under reference number 14_211RA.

Data sharing: The SPSS syntax documents detailing the steps taken to create the analysis databases from raw CPRD and HES data are available upon request from the corresponding author.

Funding: The research was funded by the National Institute for Health Research (NIHR) Biomedical Research Centre based at Imperial College Healthcare NHS Trust and Imperial College London. The views expressed are those of the authors and not necessarily those of the NHS, the NIHR or the Department of Health.

This project was also supported by the Peter Sowerby Foundation.

Competing interests: The authors have no competing interests

Ethics approval: No ethics approval was required

Patient involvement: No patients were involved in this study

Transparency declaration: The authors affirm that the manuscript is an honest, accurate, and transparent account of the study being reported; that no important aspects of the study have been omitted; and that any discrepancies from the study as planned have been explained.

For peer review only

For peer review only - http://bmjopen.bmj.com/site/about/guidelines.xhtml

Contributors: SV designed the study, created the database, analysed the data, and drafted and revised the paper. She is guarantor. GF and EM contributed to the design of the study, analysed the results and revised the draft paper. AD contributed to the design of the study and revised the draft paper. All have approved the final version for publication.

For peer review only

Exclusive license: The Corresponding Author has the right to grant on behalf of all authors and does grant on behalf of all authors, a worldwide licence to the Publishers and its licensees in perpetuity, in all forms, formats and media (whether known now or created in the future), to i) publish, reproduce, distribute, display and store the Contribution, ii) translate the Contribution into other languages, create adaptations, reprints, include within collections and create summaries, extracts and/or, abstracts of the Contribution and convert or allow conversion into any format including without limitation audio, iii) create any other derivative work(s) based in whole or part on the on the Contribution, iv) to exploit all subsidiary rights to exploit all subsidiary rights that currently exist or as may exist in the future in the Contribution, v) the inclusion of electronic links from the Contribution to third party material where-ever it may be located; and, vi) licence any third party to do any or all of the above.

A **RECORD checklist** has been completed for this study. "REporting of studies Conducted using Observational Routinely-collected Data" (RECORD) extends the STROBE guidelines to studies using administrative data.

For peer review only - http://bmjopen.bmj.com/site/about/guidelines.xhtml

REFERENCES

1. Billings J, Anderson GM, Newman LS. Recent findings on preventable hospitalizations. Health Aff (Millwood) 1996;**15**(3):239-49.
2. Bindman AB, Grumbach K, Osmond D, et al. Preventable hospitalizations and access to health care. JAMA 1995;**274**(4):305-11.
3. Billings J, Zeitel L, Lukomnik J, et al. Impact of socioeconomic status on hospital use in New York City. Health Aff (Millwood) 1993;**12**(1):162-73.
4. Caminal J, Starfield B, Sánchez E, et al. The role of primary care in preventing ambulatory care sensitive conditions. The European Journal of Public Health 2004;**14**(3):246-51.
5. Magán P, Alberquilla Á, Otero Á, et al. Hospitalizations for Ambulatory Care Sensitive Conditions and Quality of Primary Care: Their Relation With Socioeconomic and Health Care Variables in the Madrid Regional Health Service (Spain). Med Care 2011;**49**(1):17-23.
6. Menec VH, Sirski M, Attawar D, et al. Does continuity of care with a family physician reduce hospitalizations among older adults? J Health Serv Res Policy 2006;**11**(4):196-201.
7. Lambrew J, Defriese G, Carey T, et al. The Effects of Having a Regular Doctor on Access to Primary Care. Med Care 1996;**34**(2):138-51.
8. Huntley A, Lasserson D, Wye L, et al. Which features of primary care affect unscheduled secondary care use? A systematic review. BMJ Open 2014;**4**(5).
9. Laditka JN, Laditka SB, Probst JC. More May Be Better: Evidence of a Negative Relationship between Physician Supply and Hospitalization for Ambulatory Care Sensitive Conditions. Health Serv Res 2005;**40**(4):1148-66.
10. Ansari Z, Laditka JN, Laditka SB. Access to Health Care and Hospitalization for Ambulatory Care Sensitive Conditions. Med Care Res Rev 2006;**63**(6):719-41.
11. Basu J, Friedman B, Burstin H. Primary Care, HMO Enrollment, and Hospitalization for Ambulatory Care Sensitive Conditions: A New Approach. Med Care 2002;**40**(12):1260-69.
12. Macinko J, de Oliveira VB, Turci MA, et al. The Influence of Primary Care and Hospital Supply on Ambulatory Care–Sensitive Hospitalizations Among Adults in Brazil, 1999–2007. Am J Public Health 2011;**101**(10):1963-70.
13. Rizza P, Bianco A, Pavia M, et al. Preventable hospitalization and access to primary health care in an area of Southern Italy. BMC Health Serv Res 2007;**7**(1):1-8.
14. Freund T, Campbell SM, Geissler S, et al. Strategies for Reducing Potentially Avoidable Hospitalizations for Ambulatory Care–Sensitive Conditions. The Annals of Family Medicine 2013;**11**(4):363-70.
15. Probst JC, Laditka JN, Laditka SB. Association between community health center and rural health clinic presence and county-level hospitalization rates for ambulatory

For peer review only - http://bmjopen.bmj.com/site/about/guidelines.xhtml

care sensitive conditions: an analysis across eight US states. BMC Health Serv Res 2009;**9**(1):1-11.

16. Blunt I. Focus on preventable admissions - Trends in emergency admissions for ambulatory care sensitive conditions, 2001 to 2013. Quality Watch. London: The Health Foundation & Nuffield Trust, 2013.
17. Busby J, Purdy S, Hollingworth W. A systematic review of the magnitude and cause of geographic variation in unplanned hospital admission rates and length of stay for ambulatory care sensitive conditions. BMC Health Serv Res 2015;**15**(1):324.
18. Morse A. Emergency admissions to hospital: managing the demand. London: National Audit Office, 2013.
19. Berwick DM, Nolan TW, Whittington J. The Triple Aim: Care, Health, And Cost. Health Aff (Millwood) 2008;**27**(3):759-69.
20. NHS Information Centre for Health and Social Care. NHS Outcomes Framework 2014/15: Domain 2 Enhancing quality of life for people with long-term conditions - Indicator specifications. Leeds: NHS Information Centre for Health and Social Care, 2014.
21. Agency for Healthcare Research and Quality. Prevention Quality Indicators Overview. Secondary Prevention Quality Indicators Overview. http://www.qualityindicators.ahrq.gov/modules/pqi_overview.aspx.
22. Canadian Institute for Health Information. Technical Note: Ambulatory Care Sensitive Conditions (ACSCs) Hospitalization Rate. Secondary Technical Note: Ambulatory Care Sensitive Conditions (ACSCs) Hospitalization Rate. https://www.cihi.ca/en/health-system-performance/performance-reporting/indicators/technical-note-ambulatory-care-sensitiv-0.
23. Glover J, Page A, Ambrose S, et al. Atlas of Avoidable Hospitalisations in Australia: ambulatory care-sensitive conditions. Canberra: Australian Institute of Health and Welfare, 2007.
24. Solberg LI. Preventable hospital admissions: are they? Family Practice 2015;**32**(3):245-46.
25. Laditka JN, Laditka SB, Mastanduno MP. Hospital utilization for ambulatory care sensitive conditions: health outcome disparities associated with race and ethnicity. Social science & medicine 2003;**57**(8):1429-41.
26. Purdy S. Avoiding hospital admissions - What does the research evidence say? London: The Kings Fund, 2010.
27. Gibson OR, Segal L, McDermott RA. A systematic review of evidence on the association between hospitalisation for chronic disease related ambulatory care sensitive conditions and primary health care resourcing. BMC Health Serv Res 2013;**13**(1):1-13.
28. Ricketts TC, Randolph R, Howard HA, et al. Hospitalization rates as indicators of access to primary care. Health & Place 2001;**7**(1):27-38.

For peer review only - http://bmjopen.bmj.com/site/about/guidelines.xhtml

29. Sanmartin C, Khan S, Longitudinal Health Administrative Data Research team. Hospitalizations for ambulatory care sensitive conditions (ACSC): The factors that matter. Health Research Working Paper Series. Ottowa: Statistics Canada, 2011.

30. Purdy S, Griffin T, Salisbury C, et al. Ambulatory care sensitive conditions: terminology and disease coding need to be more specific to aid policy makers and clinicians. Public Health 2009;**123**(2):169-73.

31. Technology Reference Data Update Distribution Service. NHS UK Read Codes Clinical Terms Version 3. 18.0.2_20141001000001 ed. Leeds: NHS Information Centre for Health and Social Care, 2014.

32. Goddard M, Smith P. Equity of access to health care services: Theory and evidence from the UK. Social science & medicine 2001;**53**(9):1149-62.

33. IBM SPSS Statistics for Macintosh, Version 23.0 [program]. Armonk, NY: IBM Corp, 2015.

34. NHS Health and Social Care Information Centre. CCG OIS 2.6 Unplanned hospitalisation for chronic ambulatory care sensitive conditions. December 2015 ed, 2015.

35. Virnig BA, McBean M. Administrative Data for Public Health Surveillance and Planning. Annual Review of Public Health 2001;**22**(1):213-30.

36. Arah OA, Westert GP, Hurst J, et al. A conceptual framework for the OECD Health Care Quality Indicators Project. Int J Qual Health Care 2006;**18**(suppl 1):5-13.

37. Roos LL, Walld R, Uhanova J, et al. Physician Visits, Hospitalizations, and Socioeconomic Status: Ambulatory Care Sensitive Conditions in a Canadian Setting. Health Serv Res 2005;**40**(4):1167-85.

For peer review only

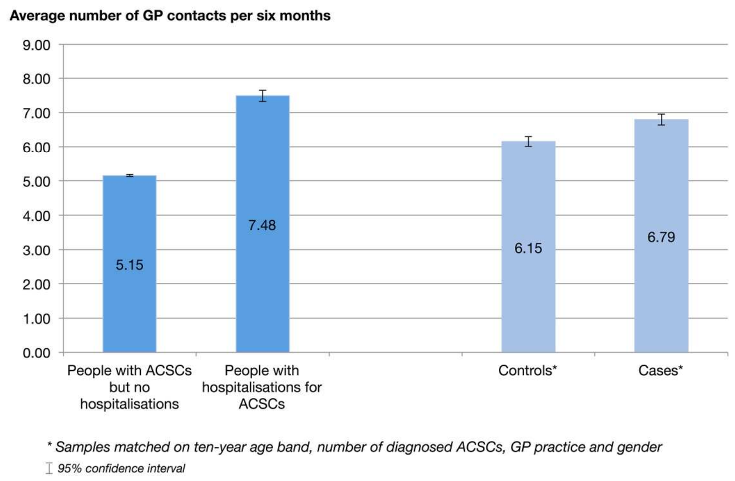

Average number of GP contacts per six months

209x148mm (300 x 300 DPI)

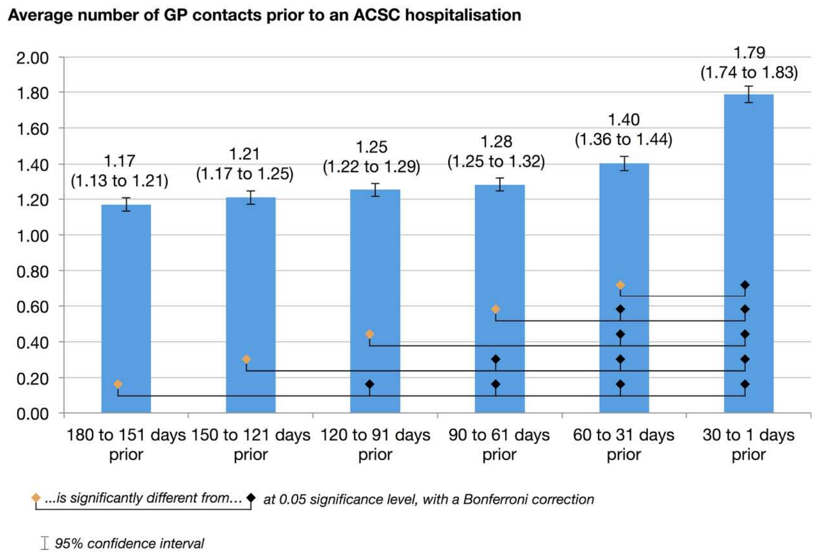

Average number of GP contacts prior to an ACSC hospitalisation

190x142mm (300 x 300 DPI)

The RECORD statement – checklist of items, extended from the STROBE statement, that should be reported in observational studies using routinely collected health data.

	Item No.	STROBE items	Location in manuscript where items are reported	RECORD items	Location in manuscript where items are reported
**Title and abstract**					
	1	(a) Indicate the study's design with a commonly used term in the title or the abstract (b) Provide in the abstract an informative and balanced summary of what was done and what was found		RECORD 1.1: The type of data used should be specified in the title or abstract. When possible, the name of the databases used should be included.<br><br>RECORD 1.2: If applicable, the geographic region and timeframe within which the study took place should be reported in the title or abstract.<br><br>RECORD 1.3: If linkage between databases was conducted for the study, this should be clearly stated in the title or abstract.	Abstract<br><br>Abstract<br><br>Abstract
**Introduction**					
Background rationale	2	Explain the scientific background and rationale for the investigation being reported			Background (paragraph 1,2)
Objectives	3	State specific objectives, including any prespecified hypotheses			Background (paragraph 3)
**Methods**					
Study Design	4	Present key elements of study design early in the paper			Methods (study design)
Setting	5	Describe the setting, locations, and relevant dates, including periods of recruitment, exposure, follow-up, and data collection			Methods (data)
Participants	6	*(a) Cohort study* - Give the eligibility criteria, and the		RECORD 6.1: The methods of study population selection (such as codes or	Methods (data)

For peer review only

		sources and methods of selection of participants. Describe methods of follow-up<br>*Case-control study* - Give the eligibility criteria, and the sources and methods of case ascertainment and control selection. Give the rationale for the choice of cases and controls<br>*Cross-sectional study* - Give the eligibility criteria, and the sources and methods of selection of participants<br><br>*(b) Cohort study* - For matched studies, give matching criteria and number of exposed and unexposed<br>*Case-control study* - For matched studies, give matching criteria and the number of controls per case		algorithms used to identify subjects) should be listed in detail. If this is not possible, an explanation should be provided.<br><br>RECORD 6.2: Any validation studies of the codes or algorithms used to select the population should be referenced. If validation was conducted for this study and not published elsewhere, detailed methods and results should be provided.<br><br>RECORD 6.3: If the study involved linkage of databases, consider use of a flow diagram or other graphical display to demonstrate the data linkage process, including the number of individuals with linked data at each stage.	 <br><br><br><br>N/A<br><br><br><br><br><br><br>N/A
Variables	7	Clearly define all outcomes, exposures, predictors, potential confounders, and effect modifiers. Give diagnostic criteria, if applicable.		RECORD 7.1: A complete list of codes and algorithms used to classify exposures, outcomes, confounders, and effect modifiers should be provided. If these cannot be reported, an explanation should be provided.	Available upon request from author as per the data sharing statement
Data sources/ measurement	8	For each variable of interest, give sources of data and details of methods of assessment (measurement).<br>Describe comparability of assessment methods if there is more than one group			Methods (design, data)
Bias	9	Describe any efforts to address potential sources of bias			N/A
Study size	10	Explain how the study size was			Methods (data)

For peer review only

		arrived at			
Quantitative variables	11	Explain how quantitative variables were handled in the analyses. If applicable, describe which groupings were chosen, and why			Methods (design, data)
Statistical methods	12	(a) Describe all statistical methods, including those used to control for confounding<br>(b) Describe any methods used to examine subgroups and interactions<br>(c) Explain how missing data were addressed<br>(d) *Cohort study* - If applicable, explain how loss to follow-up was addressed<br>*Case-control study* - If applicable, explain how matching of cases and controls was addressed<br>*Cross-sectional study* - If applicable, describe analytical methods taking account of sampling strategy<br>(e) Describe any sensitivity analyses			Methods (statistical analysis)
Data access and cleaning methods		..		RECORD 12.1: Authors should describe the extent to which the investigators had access to the database population used to create the study population.<br><br>RECORD 12.2: Authors should provide information on the data cleaning methods used in the study.	Methods (data)<br><br>Methods (data)
Linkage		..		RECORD 12.3: State whether the study included person-level, institutional-	Methods (data)

For peer review only

				level, or other data linkage across two or more databases. The methods of linkage and methods of linkage quality evaluation should be provided.	
**Results**					
Participants	13	(a) Report the numbers of individuals at each stage of the study (*e.g.*, numbers potentially eligible, examined for eligibility, confirmed eligible, included in the study, completing follow-up, and analysed)<br>(b) Give reasons for non-participation at each stage.<br>(c) Consider use of a flow diagram		RECORD 13.1: Describe in detail the selection of the persons included in the study (*i.e.,* study population selection) including filtering based on data quality, data availability and linkage. The selection of included persons can be described in the text and/or by means of the study flow diagram.	Methods (data)
Descriptive data	14	(a) Give characteristics of study participants (*e.g.*, demographic, clinical, social) and information on exposures and potential confounders<br>(b) Indicate the number of participants with missing data for each variable of interest<br>(c) *Cohort study* - summarise follow-up time (*e.g.*, average and total amount)			Results (paragraph 1)
Outcome data	15	*Cohort study* - Report numbers of outcome events or summary measures over time<br>*Case-control study* - Report numbers in each exposure category, or summary measures of exposure<br>*Cross-sectional study* - Report numbers of outcome events or summary measures			Results
Main results	16	(a) Give unadjusted estimates			Results

For peer review only

		and, if applicable, confounder-adjusted estimates and their precision (e.g., 95% confidence interval). Make clear which confounders were adjusted for and why they were included<br>(b) Report category boundaries when continuous variables were categorized<br>(c) If relevant, consider translating estimates of relative risk into absolute risk for a meaningful time period			
Other analyses	17	Report other analyses done—e.g., analyses of subgroups and interactions, and sensitivity analyses			
**Discussion**					
Key results	18	Summarise key results with reference to study objectives			Discussion (paragraph 1,2)
Limitations	19	Discuss limitations of the study, taking into account sources of potential bias or imprecision. Discuss both direction and magnitude of any potential bias		RECORD 19.1: Discuss the implications of using data that were not created or collected to answer the specific research question(s). Include discussion of misclassification bias, unmeasured confounding, missing data, and changing eligibility over time, as they pertain to the study being reported.	Discussion (paragraph 4)
Interpretation	20	Give a cautious overall interpretation of results considering objectives, limitations, multiplicity of analyses, results from similar studies, and other relevant evidence			Discussion (paragraph 5)
Generalisability	21	Discuss the generalisability (external validity) of the study results			N/A

For peer review only

Other Information					
Funding	22	Give the source of funding and the role of the funders for the present study and, if applicable, for the original study on which the present article is based			Statements (Funding)
Accessibility of protocol, raw data, and programming code		..		RECORD 22.1: Authors should provide information on how to access any supplemental information such as the study protocol, raw data, or programming code.	Statements (Data sharing)

*Reference: Benchimol EI, Smeeth L, Guttmann A, Harron K, Moher D, Petersen I, Sørensen HT, von Elm E, Langan SM, the RECORD Working Committee. The REporting of studies Conducted using Observational Routinely-collected health Data (RECORD) Statement. *PLoS Medicine* 2015; in press.

*Checklist is protected under Creative Commons Attribution (CC BY) license.

For peer review only

BMJ Open

Do hospitalisations for Ambulatory Care Sensitive Conditions reflect low access to primary care? An observational cohort study of primary care utilisation prior to hospitalisation

Journal:	*BMJ Open*
Manuscript ID	bmjopen-2016-015704.R2
Article Type:	Research
Date Submitted by the Author:	23-Jun-2017
Complete List of Authors:	Vuik, Sabine; Imperial College London, Institute of Global Health Innovation<br>Fontana, Gianluca; Imperial College London, Institute of Global Health Innovation<br>Mayer, Erik; Imperial College London, Dept. of Surgery & Cancer<br>Darzi, Ara; Imperial College London,
**Primary Subject Heading**:	Health services research
Secondary Subject Heading:	General practice / Family practice
Keywords:	PRIMARY CARE, access to care, ACSC, Quality in health care < HEALTH SERVICES ADMINISTRATION & MANAGEMENT

SCHOLARONE™
Manuscripts

For peer review only

Do hospitalisations for Ambulatory Care Sensitive Conditions reflect low access to primary care? An observational cohort study of primary care utilisation prior to hospitalisation

Sabine I Vuik, Gianluca Fontana, Erik Mayer, Ara Darzi

Sabine I Vuik, Policy Fellow, Institute of Global Health Innovation

Imperial College, 10th Floor, St Mary's Hospital, Praed Street, London, W1 2NY, UK

Gianluca Fontana, Senior Policy Fellow, Institute of Global Health Innovation

Imperial College, 10th Floor, St Mary's Hospital, Praed Street, London, W1 2NY, UK

Erik Mayer, Clinical Senior Lecturer, Department of Surgery

Imperial College, 10th Floor, St Mary's Hospital, Praed Street, London, W1 2NY, UK

Ara Darzi, Professor in Surgery, Department of Surgery & Institute of Global Health Innovation

Imperial College, 10th Floor, St Mary's Hospital, Praed Street, London, W1 2NY, UK

For peer review only

For peer review only - http://bmjopen.bmj.com/site/about/guidelines.xhtml

Correspondence to: Sabine Vuik, s.vuik@imperial.ac.uk

+44(0) 795 714 0479 (no fax available)

Room 1029, 10th Floor, QEQM building, St Mary's Hospital, Praed Street, London, W1 2NY, UK

Keywords: primary care quality, ACSC hospitalisations, integrated care, access to care

Word count (excl. title page, abstract, references, tables and figures): 2,768

For peer review only

ABSTRACT

Objectives To explore whether hospitalisations for Ambulatory Care Sensitive Conditions (ACSCs) are associated with low access to primary care

Design Observational cohort study over 2008 to 2012 using the Clinical Practice Research Datalink (CPRD) and Hospital Episode Statistics (HES) databases

Setting English primary and secondary care

Participants A random sample of 300,000 patients

Main outcome measures Emergency hospitalisation for an ACSC

Results Over the long-term, patients with ACSC hospitalisations had on average 2.33 (2.17 to 2.49) more GP contacts per six months than patients with similar conditions who did not require hospitalisation. When accounting for the number of diagnosed ACSCs, age, gender and GP practice through a nested case-control method, the difference was smaller (0.64 contacts) but still significant ($p<0.001$). People with ACSC hospitalisations also had significantly more outpatient appointments and non-ACSC emergency hospitalisations, but fewer elective day case admissions.

In the short-term analysis, measured over the six months prior to hospitalisation, patients used more GP services than on average over the five years. Cases had significantly ($p<0.001$) more primary care contacts in the six months before ACSC hospitalisations (7.12, 95% CI: 6.95 to 7.30) than their controls during the same six months (5.57, 95% CI: 5.43 to 5.72). The use of GP services increased closer to the time of hospitalisation, with a peak of 1.79 (1.74 to 1.83) contacts in the last 30 days before hospitalisation.

Conclusions This study found no evidence to support the hypothesis that low access to primary care is the main driver of ACSC hospitalisations. Other causes should also be explored to understand how to use ACSC admission rates as quality metrics, and to develop the appropriate interventions.

STRENGTHS AND LIMITATIONS OF THIS STUDY

For peer review only

For peer review only - http://bmjopen.bmj.com/site/about/guidelines.xhtml

- This study uses linked primary and secondary care data to explore whether there exists a patient-level, temporal relation between low utilisation of primary care and ACSC hospitalisations
- It considers both long term (5 years) and short term (6 months prior to hospitalisations) primary care utilisation
- To account for confounders, this study uses a nested case-control design, in addition to looking at average rates in the population
- While primary care utilisation can be considered a proxy of 'realised' access to care, it remains only a proxy and does not consider unmet needs
- This study only looks at access to primary care, and does not explore other quality aspects of primary care that may be related to ACSC hospitalisation, such as timeliness and effectiveness

For peer review only

For peer review only - http://bmjopen.bmj.com/site/about/guidelines.xhtml

BACKGROUND

Chronic ambulatory care sensitive conditions (ACSCs) are conditions like diabetes, COPD and angina that are cared for in the community by primary care providers.[1-3] With high quality primary care - which is timely,[4,5] effective,[4] continuous,[5-8] and accessible[1,9-15] - the need for hospital care for these conditions can be reduced. Therefore, high rates of emergency hospitalisations for chronic ACSCs can be an indication of lower quality primary care.[1,16,17]

In addition to providing a measure of low care quality, emergency hospitalisations for ACSC also reflect a major health system expenditure,[18] and a negative patient experience - covering all elements of the Triple Aim.[19] As a result, the rate of ACSC hospitalisation has been widely used to measure the overall quality of primary care, by national organisations in the UK, the USA, Canada and Australia.[20-23]

Despite the widespread use of these metrics, empirical evidence of their validity is limited.[24] In particular, ACSC hospitalisations are often considered a reflection of access to primary care,[1,9-15,25] but the actual relation between primary care access and hospitalisations for ACSCs remains unclear.[24,26] Various studies have found low access to primary care physicians to be related to higher rates of ACSC hospitalisation.[9-13,15,27] However, these are ecological studies using high-level proxies for primary care access, such as the density of primary care physicians in a certain region.[9-11,27] Other studies have found no relation between primary care access and ACSC hospitalisation rates.[5,28,29]

Understanding what drives hospitalisations for ACSCs is the first step in developing effective policies to address the issue. This paper aims to identify whether there is a relation between low access to primary care and ACSC hospitalisations. Using administrative data linked across primary and secondary care, it explores this relation at a

For peer review only - http://bmjopen.bmj.com/site/about/guidelines.xhtml

patient-level. It considers both long-term access to primary care, reflecting the long-term management of the condition, and access in the six months prior to a hospitalisation.

METHODS

Study overview

Using a retrospective cohort study, this paper analyses the relation between primary care utilisation and hospitalisations for ACSCs at the patient-level. In addition to exploring population-level rates, matched case-control samples were selected and compared to adjust for a range of confounders. The study looks at both long-term utilisation, as measured over five years, and utilisation directly prior to ACSC hospitalisation, as measured over the six months preceding hospitalisation.

To understand the long-term levels of primary care utilisation for patients with ACSC hospitalisations, the average number of GP contacts per six months was calculated from five years of data. To contextualise this utilisation rate and determine whether it is low or high, it was compared to the utilisation rates of two reference populations: patients without any diagnosed ACSCs, and patients with diagnosed ACSCs but no qualifying emergency hospitalisations. *(Note that, for simplicity, the second group is called 'patients with ACSCs but no hospitalisations'. This only refers to qualifying emergency admissions for ACSCs as defined below. Patients in this group may still have had elective hospitalisations, or hospitalisations for non-ACSC causes).*

To explore whether a temporary lack of primary care contributes to ACSC hospitalisations, primary care utilisation in the six months directly prior to the hospitalisation was also calculated. In addition, utilisation rates for six consecutive 30-day intervals were calculated to study changes in care access leading up to an ACSC hospitalisation. These retrospective

For peer review only - http://bmjopen.bmj.com/site/about/guidelines.xhtml

analyses were done at the ACSC hospitalisation level rather than the patient level by identifying the six months of GP utilisation prior to each individual ACSC hospitalisation. All hospitalisations were included, even if they occurred in the same person, or if their six months of prior activity overlapped with another ACSC hospitalisation for that patient.

Data sources and study sample

Using the Clinical Practice Research Datalink (CPRD) and Hospital Episode Statistics (HES), a database containing a random sample of 300,000 English patients and their five-year (2008-2012) primary care and secondary care utilisation was created. Patients were eligible for inclusion in the study if they were registered with a CPRD participating GP throughout the entire observation period, and if their primary care data could be linked to their secondary care data in HES. Linkage of the data was done by CPRD, and required consent and the availability of matching NHS number and other personal details (note: this information was not available to the authors, who only had anonymous data). The sample size was set at 300,000, which is the maximum sample size CPRD ordinarily allows.

Main outcome measures

The main measures in this study were hospitalisations for ACSCs, and primary care utilisation.

Hospitalisations for ACSC admissions: In the HES data, ACSC hospitalisations were identified following the definitions below. To ensure six months of primary care activity was available preceding each ACSC hospitalisation, only hospitalisations in the final 4.5 years were included. All ACSC hospitalisations were included, even if they were for the same person or within the six-month period prior to another ACSC hospitalisation.

For peer review only - http://bmjopen.bmj.com/site/about/guidelines.xhtml

The definition of ACSCs generally includes both chronic and acute conditions.[30] This study focuses on chronic ACSCs only, as it is interested in long-term disease management in the primary care setting. In addition, it allows a comparator group to be created consisting of people with similar chronic conditions but no hospitalisations. A wide variety of ACSC conditions and coding practices exists.[30] This research defines ACSCs according to the ICD-10 codes used in the NHS England Outcomes Framework, as these are specific to English administrative data.[20]

The diagnosis codes for ACSC hospitalisations were also used to identify patients who have been diagnosed with those conditions in the 300,000 patients sample. Patients' morbidity profiles were based on a combination of both the HES and CPRD dataset. Without data outside the five-year study period, we were unable to identify whether a diagnosis code at a certain time point is a newly onset disease, or reflects treatment for an existing condition. Therefore, no temporal analysis was possible, and a diagnosis at any time point was treated as a positive flag for that condition. To be able to find ACSC diagnoses in the CPRD database, the ICD-10 definitions had to be translated to READ codes, which are used in primary care. A mapping created by the NHS Health and Social Care Information Centre was used for this purpose.[31]

Care utilisation: This study aims to measure access to care. While it is difficult to measure 'potential' care access, which includes unmet needs, 'realised' care access can be measured in terms of care utilisation rates.[32] Therefore, utilisation rates are used as a proxy for access. It is important to note that this metric measures the patient's frequency of contact with healthcare providers, but it does not consider other aspects of access such as the length of the contact or instances where a patient is unable to get an appointment.

For peer review only

Utilisation rates were measured for primary care by counting the number of GP contacts, including surgery and clinic attendances, home visits and telephone calls. No distinction was made between different types of practice staff, and care could have been provided by practice nurses and other qualified staff. Therefore, a 'GP contact' should be interpreted as a contact with the GP practice rather than a specific GP.

Statistical analysis

Using SPSS Statistics version 23,[33] descriptive statistics, including average utilisation rates, were calculated for the overall population and the three population groups. Independent t-tests were used to compare the utilisation rates in the population with ACSCs hospitalisations to the population with ACSCs but no hospitalisations.

To adjust for timing differences as well as confounders such as age and ACSC counts, a nested case-control method was used. Using the FUZZY extension in SPSS, patients with an ACSC hospitalisation were matched to a control, based on gender, number of diagnosed ACSCs, GP practice (to account for provider-level differences) and ten-year age bands. The long-term primary care utilisation rates, as well as utilisation rates of other care settings, were compared using independent t-tests between the cases and controls. For the utilisation prior to ACSC hospitalisation, the same six-month time period preceding the hospitalisation of the cases was used for the controls, to account for seasonal or other timing differences.

To analyse the change in utilisation rate leading up to an ACSC hospitalisation, a general linear model for repeated measures was used with the utilisation rates for the six 30-day intervals as the response variable. These intervals were compared pair-wise as well, adjusting the significance level for multiple tests using a Bonferroni correction.

For peer review only - http://bmjopen.bmj.com/site/about/guidelines.xhtml

For peer review only

RESULTS

In the database, 7,467 hospitalisations for ACSCs were identified for the sample of 300,000 people over 4.5 years. This equates to an admission rate of 553 per 100,000 people per year, which is in line with crude ACSC admission rates for regions in England in 2014/15, which range from 137 to 1,384 per 100,000.[34]

Nearly one-third (70,369) of people in the population had diagnoses for one or more ACSCs (see table 1). Of these people, 7% (5,190) had one or more hospitalisations for their ACSCs, with 4,017 people having one ACSC hospitalisation during the 4.5 years, and 1,173 people more than one.

Table 1: Sample characteristics for the three population groups

	**People with no ACSCs**	**People with ACSCs but no hospitalisations**	**People with hospitalisations for ACSCs**	**Total population**
N	229,631	65,179	5,190	**300,000**
Age (average)	41	58	63	**45**
Gender				
Female (%)	50%	53%	50%	**51%**
Male (%)	50%	47%	50%	**49%**
Number of diagnosed ACSCs (average)	0	1.47	2.60	**0.36**
Number of hospitalisations for ACSCs over 4.5 years (average)	0	0	1.44	**0.02**

For peer review only - http://bmjopen.bmj.com/site/about/guidelines.xhtml

For 76% (3,957) of all people with an ACSC hospitalisation an exactly matched control was found (see table 2). This subgroup accounted for 70% of all ACSC hospitalisations in the database. While a ten-year age band was used to match people rather than exact age, there was no significant (p=0.62) age difference between the two groups.

Table 2: Sample characteristics of the nested cases and controls

	**Cases**	**Controls**
N	3,957	3,957
Age (average)	62.02	62.27
Gender		
Female (%)	50%	50%
Male (%)	50%	50%
Number of diagnosed ACSCs (average)	2.13	2.13

Over the full study period of five years, patients with ACSC hospitalisations had significantly more (2.33, p<0.001) GP contacts per six months (7.48, 95% CI: 7.32 to 7.64) than people with similar conditions who did not require hospitalisation (5.15, 95% CI: 5.12 to 5.18) (see figure 1). The nested case-control method adjusts for the number of diagnosed ACSCs and other confounders. While the difference between these two groups was smaller, people with ACSC hospitalisations still had significantly more (0.64, p<0.001) GP contacts than their controls.

Since the utilisation directly prior to a hospitalisation is measured over only six months, there may be seasonal differences for this metric. Therefore, primary care utilisation prior to hospitalisation in the cases was compared to utilisation in the controls over the same six-month time period. People with ACSC hospitalisations had significantly more (1.55,

For peer review only - http://bmjopen.bmj.com/site/about/guidelines.xhtml

For peer review only

p<0.001) primary care contacts in the six months before hospitalisations (7.12, 95% CI: 6.95 to 7.30) than their controls during the same six months (5.57, 95% CI: 5.43 to 5.72).

To explore differences in primary care use leading up to ACSC hospitalisations, the utilisation rates of six consecutive 30-day intervals were calculated (see figure 2). GP contacts were skewed towards the months closer to the hospitalisation date. A repeated measures general linear model showed that the mean number of GP contacts is significant different (p<0.001) across the six 30-day intervals. Pairwise comparisons show that the peak in GP contacts in the final 30 days prior to hospitalisation is significantly different from each of the five preceding months. *(Note: the sum of these intervals (8.11) is slightly less than the six-month utilisation (8.19), as the latter is calculated over 182 days rather than 180 to reflect 6 calendar months, allowing comparison to the 5-year utilisation rates).*

DISCUSSION

The primary objective of this study was to explore whether low access to primary care is related to hospitalisations for ACSCs. No evidence was found of low primary care utilisation either over the long term, which could affect the on-going management of ACSCs, or in the short term, prior to an ACSC hospitalisation. The results show that, over five years, patients with ACSC hospitalisations in fact use more GP services compared to other patients with the same number of diagnosed ACSCs, age, gender and GP practice.

Neither does there appear to be a temporary drop in utilisation. In the six months immediately prior to an ACSC hospitalisation, utilisation is higher than the five-year average. People with ACSC hospitalisations use more primary care than matched controls over the same six-month period. In addition, the average utilisation rate increased every month nearer to the hospitalisation, with a significant peak in the last 30 days prior to

For peer review only - http://bmjopen.bmj.com/site/about/guidelines.xhtml

hospitalisation. Overall, this research finds no evidence that low access to GP services is a key driver of ACSC hospitalisation.

This study found a statistically significant relation between primary care utilisation and ACSC hospitalisations, where the utilisation rate increases prior to hospitalisation. Future research should explore whether this correlation can be used to improve risk models. Predictive models could be developed that predict ACSC hospitalisations based on a change in GP utilisation, allowing preventive interventions to be delivered.

An important strength of this study is the use of linked, administrative data at the patient level. Rather than using population-level proxies for access, as has been done in previous studies on this topic,[9-11,27] this study linked primary care utilisation and ACSC hospitalisations at the patient level. In addition, it considered both long-term care use and care utilisation immediately preceding an ACSC hospitalisation. Using this approach, the direct, temporal relation between a patient's access to primary care and ACSC hospitalisation could be explored.

The research has a number of limitations, in addition to the well-documented general limitations associated with using administrative healthcare data.[35] Firstly, while utilisation approximates access to care at the patient level, it is still only a proxy. No differentiation can be made between low utilisation due to limited access to services, and other causes such as patients not seeking care. In addition, this proxy does not take into account any unmet needs. Utilisation rates for patients with ACSC hospitalisations were in line with those of people with similar disease profiles, but their actual need for care may have been even higher - as suggested in their eventual hospitalisation. The fact that ACSC hospitalisations are not associated with lower access to primary care does not preclude that more primary care could prevent them.

For peer review only - http://bmjopen.bmj.com/site/about/guidelines.xhtml

For peer review only

This is a crucial limitation of this research, and of any similar analyses based on administrative data. To fully understand what drives ACSC hospitalisations at the individual patient-level, case note reviews or interviews are needed. Administrative data only covers actualised care. A case note review study could verify the results from this research, as well as the overall approach of using administrative data to measure care needs.

Secondly, this paper only explores the relation between low access to primary care and ACSC hospitalisations. ACSC hospitalisation rates are used as a high-level measure of primary care, and can been linked to many other aspects of care quality, such as timeliness,[4,5] effectiveness,[4] continuity,[5-7] safety and appropriateness.[36] Further research is needed to understand the specific and relative impact of these factors on ACSC hospitalisations.

Thirdly, there remains the possibility of confounding. While a nested case-control method was used to account for diagnosed ACSCs, age, gender and provider differences, other factors such as urban/rural environments, ethnicity[25] and socio-economic status[5,37] could be influencing the results. While matching on GP practice partially mitigates these issues due to patients living in the same geographic area, there remains room for confounding.

In addition, cases were matched based on the number of ACSCs rather than specific conditions - limiting the comparability of the cases and controls. This approach was chosen to ensure more matches could be found, and to create a quantitative measure of health status. Nevertheless, health status remains an important potential confounder, particularly due to its direct impact on care utilisation. This is another area where case reviews would provide a level of detail that is not available in administrative data.

For peer review only - http://bmjopen.bmj.com/site/about/guidelines.xhtml
For peer review only

The cases for which a matched control was found had slightly less primary care activity and ACSC hospitalisations than the overall population with ACSC hospitalisations. This could be due to a lack of matched controls for extreme cases with a very high age or ACSCs count, who could also be expected to use more care. The exclusion of extreme cases is a limitation of the methods used in this study, and there is an opportunity for future research to explore what drives ACSC hospitalisations in this high-needs group.

The results presented here have important implications for clinicians and policymakers. This study provides no evidence that, as is commonly believed, low access to primary care is a leading cause of ACSC hospitalisations. This would mean that ACSC hospitalisation rates, while informative, should not be used specifically as indicators of access to primary care.

More research is needed into new models of care to meet the needs of patients with ACSC hospitalisations. Policymakers and clinicians trying to reduce emergency hospitalisation should consider policies that focus on a variety of aspects of primary care, including effectiveness and continuity of care, rather than focusing on access alone. Tailored interventions aimed at high-risk patients should also be explored, including remote support and observational beds.

CONCLUSION

This research found no patient-level, temporal relation between low utilisation of primary care services and hospitalisations for ACSCs. While there may be other aspects of primary care quality that impact ACSC hospitalisation rates, there is no evidence that low access to care is a key driver. More research is needed into the actual causes of ACCS hospitalisations, across care settings, to allow accurate interpretation of ACSC hospitalisation rates and to develop effective interventions.

For peer review only

FIGURE LEGENDS

Figure 1: Average number of GP contacts per six months

Figure 2: Average number of GP contacts prior to an ACSC hospitalisation

For peer review only

For peer review only - http://bmjopen.bmj.com/site/about/guidelines.xhtml

STATEMENTS

Database: This study is based on data from the Clinical Practice Research Datalink obtained under license from the UK Medicines and Healthcare Products Regulatory Agency. However, the interpretation and conclusions contained in the study are those of the authors alone. The study was approved under reference number 14_211RA.

Data sharing: The SPSS syntax documents detailing the steps taken to create the analysis databases from raw CPRD and HES data are available upon request from the corresponding author.

Funding: The research was funded by the National Institute for Health Research (NIHR) Biomedical Research Centre based at Imperial College Healthcare NHS Trust and Imperial College London. The views expressed are those of the authors and not necessarily those of the NHS, the NIHR or the Department of Health.

This project was also supported by the Peter Sowerby Foundation.

Competing interests: The authors have no competing interests

Ethics approval: No ethics approval was required

Patient involvement: No patients were involved in this study

Transparency declaration: The authors affirm that the manuscript is an honest, accurate, and transparent account of the study being reported; that no important aspects of the study have been omitted; and that any discrepancies from the study as planned have been explained.

For peer review only

Contributors: SV designed the study, created the database, analysed the data, and drafted and revised the paper. She is guarantor. GF and EM contributed to the design of the study, analysed the results and revised the draft paper. AD contributed to the design of the study and revised the draft paper. All have approved the final version for publication.

Exclusive license: The Corresponding Author has the right to grant on behalf of all authors and does grant on behalf of all authors, a worldwide licence to the Publishers and its licensees in perpetuity, in all forms, formats and media (whether known now or created in the future), to i) publish, reproduce, distribute, display and store the Contribution, ii) translate the Contribution into other languages, create adaptations, reprints, include within collections and create summaries, extracts and/or, abstracts of the Contribution and convert or allow conversion into any format including without limitation audio, iii) create any other derivative work(s) based in whole or part on the on the Contribution, iv) to exploit all subsidiary rights to exploit all subsidiary rights that currently exist or as may exist in the future in the Contribution, v) the inclusion of electronic links from the Contribution to third party material where-ever it may be located; and, vi) licence any third party to do any or all of the above.

A **RECORD checklist** has been completed for this study. "REporting of studies Conducted using Observational Routinely-collected Data" (RECORD) extends the STROBE guidelines to studies using administrative data.

For peer review only

REFERENCES

1. Billings J, Anderson GM, Newman LS. Recent findings on preventable hospitalizations. Health Aff (Millwood) 1996;**15**(3):239-49.

2. Bindman AB, Grumbach K, Osmond D, et al. Preventable hospitalizations and access to health care. JAMA 1995;**274**(4):305-11.

3. Billings J, Zeitel L, Lukomnik J, et al. Impact of socioeconomic status on hospital use in New York City. Health Aff (Millwood) 1993;**12**(1):162-73.

4. Caminal J, Starfield B, Sánchez E, et al. The role of primary care in preventing ambulatory care sensitive conditions. The European Journal of Public Health 2004;**14**(3):246-51.

5. Magán P, Alberquilla Á, Otero Á, et al. Hospitalizations for Ambulatory Care Sensitive Conditions and Quality of Primary Care: Their Relation With Socioeconomic and Health Care Variables in the Madrid Regional Health Service (Spain). Med Care 2011;**49**(1):17-23.

6. Menec VH, Sirski M, Attawar D, et al. Does continuity of care with a family physician reduce hospitalizations among older adults? J Health Serv Res Policy 2006;**11**(4):196-201.

7. Lambrew J, Defriese G, Carey T, et al. The Effects of Having a Regular Doctor on Access to Primary Care. Med Care 1996;**34**(2):138-51.

8. Huntley A, Lasserson D, Wye L, et al. Which features of primary care affect unscheduled secondary care use? A systematic review. BMJ Open 2014;**4**(5).

9. Laditka JN, Laditka SB, Probst JC. More May Be Better: Evidence of a Negative Relationship between Physician Supply and Hospitalization for Ambulatory Care Sensitive Conditions. Health Serv Res 2005;**40**(4):1148-66.

10. Ansari Z, Laditka JN, Laditka SB. Access to Health Care and Hospitalization for Ambulatory Care Sensitive Conditions. Med Care Res Rev 2006;**63**(6):719-41.

11. Basu J, Friedman B, Burstin H. Primary Care, HMO Enrollment, and Hospitalization for Ambulatory Care Sensitive Conditions: A New Approach. Med Care 2002;**40**(12):1260-69.

12. Macinko J, de Oliveira VB, Turci MA, et al. The Influence of Primary Care and Hospital Supply on Ambulatory Care–Sensitive Hospitalizations Among Adults in Brazil, 1999–2007. Am J Public Health 2011;**101**(10):1963-70.

13. Rizza P, Bianco A, Pavia M, et al. Preventable hospitalization and access to primary health care in an area of Southern Italy. BMC Health Serv Res 2007;**7**(1):1-8.

14. Freund T, Campbell SM, Geissler S, et al. Strategies for Reducing Potentially Avoidable Hospitalizations for Ambulatory Care–Sensitive Conditions. The Annals of Family Medicine 2013;**11**(4):363-70.

15. Probst JC, Laditka JN, Laditka SB. Association between community health center and rural health clinic presence and county-level hospitalization rates for ambulatory

For peer review only

care sensitive conditions: an analysis across eight US states. BMC Health Serv Res 2009;**9**(1):1-11.

16. Blunt I. Focus on preventable admissions - Trends in emergency admissions for ambulatory care sensitive conditions, 2001 to 2013. Quality Watch. London: The Health Foundation & Nuffield Trust, 2013.
17. Busby J, Purdy S, Hollingworth W. A systematic review of the magnitude and cause of geographic variation in unplanned hospital admission rates and length of stay for ambulatory care sensitive conditions. BMC Health Serv Res 2015;**15**(1):324.
18. Morse A. Emergency admissions to hospital: managing the demand. London: National Audit Office, 2013.
19. Berwick DM, Nolan TW, Whittington J. The Triple Aim: Care, Health, And Cost. Health Aff (Millwood) 2008;**27**(3):759-69.
20. NHS Information Centre for Health and Social Care. NHS Outcomes Framework 2014/15: Domain 2 Enhancing quality of life for people with long-term conditions - Indicator specifications. Leeds: NHS Information Centre for Health and Social Care, 2014.
21. Agency for Healthcare Research and Quality. Prevention Quality Indicators Overview. Secondary Prevention Quality Indicators Overview. http://www.qualityindicators.ahrq.gov/modules/pqi_overview.aspx.
22. Canadian Institute for Health Information. Technical Note: Ambulatory Care Sensitive Conditions (ACSCs) Hospitalization Rate. Secondary Technical Note: Ambulatory Care Sensitive Conditions (ACSCs) Hospitalization Rate. https://www.cihi.ca/en/health-system-performance/performance-reporting/indicators/technical-note-ambulatory-care-sensitiv-0.
23. Glover J, Page A, Ambrose S, et al. Atlas of Avoidable Hospitalisations in Australia: ambulatory care-sensitive conditions. Canberra: Australian Institute of Health and Welfare, 2007.
24. Solberg LI. Preventable hospital admissions: are they? Family Practice 2015;**32**(3):245-46.
25. Laditka JN, Laditka SB, Mastanduno MP. Hospital utilization for ambulatory care sensitive conditions: health outcome disparities associated with race and ethnicity. Social science & medicine 2003;**57**(8):1429-41.
26. Purdy S. Avoiding hospital admissions - What does the research evidence say? London: The Kings Fund, 2010.
27. Gibson OR, Segal L, McDermott RA. A systematic review of evidence on the association between hospitalisation for chronic disease related ambulatory care sensitive conditions and primary health care resourcing. BMC Health Serv Res 2013;**13**(1):1-13.
28. Ricketts TC, Randolph R, Howard HA, et al. Hospitalization rates as indicators of access to primary care. Health & Place 2001;**7**(1):27-38.

For peer review only

For peer review only - http://bmjopen.bmj.com/site/about/guidelines.xhtml

29. Sanmartin C, Khan S, Longitudinal Health Administrative Data Research team. Hospitalizations for ambulatory care sensitive conditions (ACSC): The factors that matter. Health Research Working Paper Series. Ottowa: Statistics Canada, 2011.
30. Purdy S, Griffin T, Salisbury C, et al. Ambulatory care sensitive conditions: terminology and disease coding need to be more specific to aid policy makers and clinicians. Public Health 2009;**123**(2):169-73.
31. Technology Reference Data Update Distribution Service. NHS UK Read Codes Clinical Terms Version 3. 18.0.2_20141001000001 ed. Leeds: NHS Information Centre for Health and Social Care, 2014.
32. Goddard M, Smith P. Equity of access to health care services: Theory and evidence from the UK. Social science & medicine 2001;**53**(9):1149-62.
33. IBM SPSS Statistics for Macintosh, Version 23.0 [program]. Armonk, NY: IBM Corp, 2015.
34. NHS Health and Social Care Information Centre. CCG OIS 2.6 Unplanned hospitalisation for chronic ambulatory care sensitive conditions. December 2015 ed, 2015.
35. Virnig BA, McBean M. Administrative Data for Public Health Surveillance and Planning. Annual Review of Public Health 2001;**22**(1):213-30.
36. Arah OA, Westert GP, Hurst J, et al. A conceptual framework for the OECD Health Care Quality Indicators Project. Int J Qual Health Care 2006;**18**(suppl 1):5-13.
37. Roos LL, Walld R, Uhanova J, et al. Physician Visits, Hospitalizations, and Socioeconomic Status: Ambulatory Care Sensitive Conditions in a Canadian Setting. Health Serv Res 2005;**40**(4):1167-85.

For peer review only

For peer review only - http://bmjopen.bmj.com/site/about/guidelines.xhtml

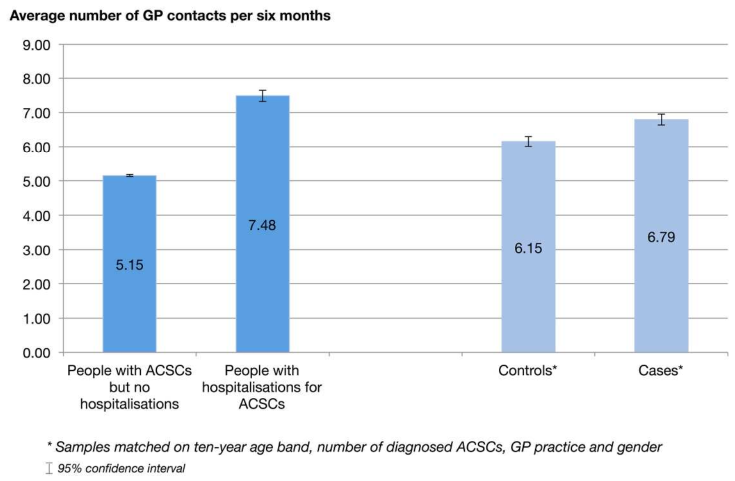

Average number of GP contacts per six months

209x148mm (300 x 300 DPI)

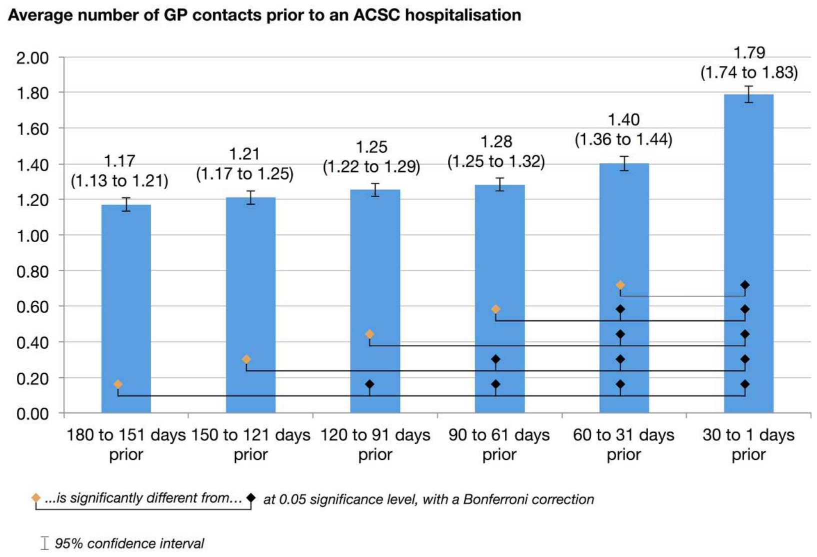

Average number of GP contacts prior to an ACSC hospitalisation

190x142mm (300 x 300 DPI)

The RECORD statement – checklist of items, extended from the STROBE statement, that should be reported in observational studies using routinely collected health data.

	Item No.	STROBE items	Location in manuscript where items are reported	RECORD items	Location in manuscript where items are reported
**Title and abstract**					
	1	(a) Indicate the study's design with a commonly used term in the title or the abstract (b) Provide in the abstract an informative and balanced summary of what was done and what was found		RECORD 1.1: The type of data used should be specified in the title or abstract. When possible, the name of the databases used should be included.<br><br>RECORD 1.2: If applicable, the geographic region and timeframe within which the study took place should be reported in the title or abstract.<br><br>RECORD 1.3: If linkage between databases was conducted for the study, this should be clearly stated in the title or abstract.	Abstract<br><br>Abstract<br><br>Abstract
**Introduction**					
Background rationale	2	Explain the scientific background and rationale for the investigation being reported			Background (paragraph 1,2)
Objectives	3	State specific objectives, including any prespecified hypotheses			Background (paragraph 3)
**Methods**					
Study Design	4	Present key elements of study design early in the paper			Methods (study design)
Setting	5	Describe the setting, locations, and relevant dates, including periods of recruitment, exposure, follow-up, and data collection			Methods (data)
Participants	6	*(a) Cohort study* - Give the eligibility criteria, and the		RECORD 6.1: The methods of study population selection (such as codes or	Methods (data)

For peer review only

		sources and methods of selection of participants. Describe methods of follow-up<br>*Case-control study* - Give the eligibility criteria, and the sources and methods of case ascertainment and control selection. Give the rationale for the choice of cases and controls<br>*Cross-sectional study* - Give the eligibility criteria, and the sources and methods of selection of participants<br><br>*(b) Cohort study* - For matched studies, give matching criteria and number of exposed and unexposed<br>*Case-control study* - For matched studies, give matching criteria and the number of controls per case		algorithms used to identify subjects) should be listed in detail. If this is not possible, an explanation should be provided.<br><br>RECORD 6.2: Any validation studies of the codes or algorithms used to select the population should be referenced. If validation was conducted for this study and not published elsewhere, detailed methods and results should be provided.<br><br>RECORD 6.3: If the study involved linkage of databases, consider use of a flow diagram or other graphical display to demonstrate the data linkage process, including the number of individuals with linked data at each stage.	 <br><br><br>N/A<br><br><br><br><br><br><br>N/A
Variables	7	Clearly define all outcomes, exposures, predictors, potential confounders, and effect modifiers. Give diagnostic criteria, if applicable.		RECORD 7.1: A complete list of codes and algorithms used to classify exposures, outcomes, confounders, and effect modifiers should be provided. If these cannot be reported, an explanation should be provided.	Available upon request from author as per the data sharing statement
Data sources/ measurement	8	For each variable of interest, give sources of data and details of methods of assessment (measurement).<br>Describe comparability of assessment methods if there is more than one group			Methods (design, data)
Bias	9	Describe any efforts to address potential sources of bias			N/A
Study size	10	Explain how the study size was			Methods (data)

For peer review only

		arrived at			
Quantitative variables	11	Explain how quantitative variables were handled in the analyses. If applicable, describe which groupings were chosen, and why			Methods (design, data)
Statistical methods	12	(a) Describe all statistical methods, including those used to control for confounding<br>(b) Describe any methods used to examine subgroups and interactions<br>(c) Explain how missing data were addressed<br>(d) *Cohort study* - If applicable, explain how loss to follow-up was addressed<br>*Case-control study* - If applicable, explain how matching of cases and controls was addressed<br>*Cross-sectional study* - If applicable, describe analytical methods taking account of sampling strategy<br>(e) Describe any sensitivity analyses			Methods (statistical analysis)
Data access and cleaning methods		..		RECORD 12.1: Authors should describe the extent to which the investigators had access to the database population used to create the study population.<br><br>RECORD 12.2: Authors should provide information on the data cleaning methods used in the study.	Methods (data)<br><br>Methods (data)
Linkage		..		RECORD 12.3: State whether the study included person-level, institutional-	Methods (data)

For peer review only

				level, or other data linkage across two or more databases. The methods of linkage and methods of linkage quality evaluation should be provided.	
**Results**					
Participants	13	(a) Report the numbers of individuals at each stage of the study (*e.g.*, numbers potentially eligible, examined for eligibility, confirmed eligible, included in the study, completing follow-up, and analysed)<br>(b) Give reasons for non-participation at each stage.<br>(c) Consider use of a flow diagram		RECORD 13.1: Describe in detail the selection of the persons included in the study (*i.e.,* study population selection) including filtering based on data quality, data availability and linkage. The selection of included persons can be described in the text and/or by means of the study flow diagram.	Methods (data)
Descriptive data	14	(a) Give characteristics of study participants (*e.g.*, demographic, clinical, social) and information on exposures and potential confounders<br>(b) Indicate the number of participants with missing data for each variable of interest<br>(c) *Cohort study* - summarise follow-up time (*e.g.*, average and total amount)			Results (paragraph 1)
Outcome data	15	*Cohort study* - Report numbers of outcome events or summary measures over time<br>*Case-control study* - Report numbers in each exposure category, or summary measures of exposure<br>*Cross-sectional study* - Report numbers of outcome events or summary measures			Results
Main results	16	(a) Give unadjusted estimates			Results

For peer review only

		and, if applicable, confounder-adjusted estimates and their precision (e.g., 95% confidence interval). Make clear which confounders were adjusted for and why they were included<br>(b) Report category boundaries when continuous variables were categorized<br>(c) If relevant, consider translating estimates of relative risk into absolute risk for a meaningful time period			
Other analyses	17	Report other analyses done—e.g., analyses of subgroups and interactions, and sensitivity analyses			
**Discussion**					
Key results	18	Summarise key results with reference to study objectives			Discussion (paragraph 1,2)
Limitations	19	Discuss limitations of the study, taking into account sources of potential bias or imprecision. Discuss both direction and magnitude of any potential bias		RECORD 19.1: Discuss the implications of using data that were not created or collected to answer the specific research question(s). Include discussion of misclassification bias, unmeasured confounding, missing data, and changing eligibility over time, as they pertain to the study being reported.	Discussion (paragraph 4)
Interpretation	20	Give a cautious overall interpretation of results considering objectives, limitations, multiplicity of analyses, results from similar studies, and other relevant evidence			Discussion (paragraph 5)
Generalisability	21	Discuss the generalisability (external validity) of the study results			N/A

For peer review only

**Other Information**					
Funding	22	Give the source of funding and the role of the funders for the present study and, if applicable, for the original study on which the present article is based			Statements (Funding)
Accessibility of protocol, raw data, and programming code		..		RECORD 22.1: Authors should provide information on how to access any supplemental information such as the study protocol, raw data, or programming code.	Statements (Data sharing)

*Reference: Benchimol EI, Smeeth L, Guttmann A, Harron K, Moher D, Petersen I, Sørensen HT, von Elm E, Langan SM, the RECORD Working Committee. The REporting of studies Conducted using Observational Routinely-collected health Data (RECORD) Statement. *PLoS Medicine* 2015; in press.

*Checklist is protected under Creative Commons Attribution (CC BY) license.

For peer review only
